# Current Trends in Nanomaterials for Metal Oxide-Based Conductometric Gas Sensors: Advantages and Limitations—Part 2: Porous 2D Nanomaterials

**DOI:** 10.3390/nano13020237

**Published:** 2023-01-05

**Authors:** Ghenadii Korotcenkov, Valeri P. Tolstoy

**Affiliations:** 1 Department of Physics and Engineering, Moldova State University, 2009 Chisinau, Moldova; 2 Institute of Chemistry, Saint Petersburg State University, Saint Petersburg 198504, Russia

**Keywords:** nanoflakes, nanosheets, nanoplates, nanoflowers, porosification, annealing, phase transition, nanoholes, sensor response, rate of response

## Abstract

This article discusses the features of the synthesis and application of porous two-dimensional nanomaterials in developing conductometric gas sensors based on metal oxides. It is concluded that using porous 2D nanomaterials and 3D structures based on them is a promising approach to improving the parameters of gas sensors, such as sensitivity and the rate of response. The limitations that may arise when using 2D structures in gas sensors intended for the sensor market are considered.

## 1. Introduction

Metal oxide nanomaterials with 1D (nanotubes, nanobelts, nanowires) and 2D structures (nanoflakes (NFs), nanoplates, and nanosheets (NSs)) are increasingly used in the development of conductometric gas sensors. Studies have shown that the unique properties of such structures make it possible to optimize the main parameters of gas sensors, such as sensitivity and rate of response [1,2,3,4,5]. In [5,6,7], a detailed consideration of the manufacturing features of gas sensors based on 1D and 2D structures and nanofibers was carried out, and the advantages and disadvantages of using such structures in this area were shown. In this article, we will continue the analysis of nanomaterials suitable for the development of gas sensors and focus on the consideration of porous 2D structures. At that, by porosity, we will understand not the traditionally considered porosity of the gas-sensitive layer formed by 2D structures but the porosity of nanoflakes and nanosheets, i.e., the presence of nanoholes in them. Some reviews about porous 2D materials and their applications have been published [8,9,10,11]. However, it should be noted that there is still a lack of a comprehensive review of the use of porous two-dimensional metal oxides for gas sensors, which is critical for the further design and manufacture of a new generation of solid-state gas sensors.

This review is organized as follows: the advantages of 2D nanomaterials for the design of gas sensors are commented on in Section 2. Then, in Section 3, we consider the limitations of developing gas sensors based on 2D nanomaterials. In Section 4, we analyze the advantages of porous 2D nanostructures for applications in gas sensors, while Section 5 discusses approaches that can be used to fabricate porous 2D structures. As regards the gas-sensitive characteristics of conductometric gas sensors based on two-dimensional structures, Section 6 and Section 7 are devoted to these issues. In Section 6, attention is focused on gas sensors using 2D structures, and in Section 7, on sensors developed on the basis of 3D nanostructures.

It should be noted that in this review, we will not consider the mechanism of gas sensitivity of metal oxide with 2D and 3D structures since this mechanism is no different from the sensitivity mechanism of conventional metal oxide gas sensors and gas sensors based on 1D nanomaterials. It has been described in numerous published articles, reviews, and books [11,12,13,14]. There is no point in repeating them. It is only important to recall that the sensory response of conductometric gas sensors is controlled by such processes as adsorption or chemisorption, desorption, diffusion, catalysis, and charge transfer, in which sensing material acts as a charge acceptor or donor with respect to the adsorbed gas molecule. As a result of these processes, an increase or decrease in the conductivity of the metal oxides occurs. The basic requirements for metal oxides to achieve maximum sensory response are described in sufficient detail in [15].

## 2. Advantages of 2D Nanomaterials for Gas Sensor Design

Korotcenkov [5] highlighted the following advantages of 2D nanostructures, such as nanosheets, nanoplates, and nanoflakes, for developing gas sensors:

- First, 2D-layered nanomaterials, such as 1D nanomaterials, possess a large surface area and high surface-to-volume ratio, allowing more atoms of these materials to interact with the atmosphere. This is especially important for gas sensors, as such structure of the gas-sensitive material facilitates surface reactions such as adsorption, chemisorption, and catalysis-controlling the gas-sensing effects [15,16].
- Second, the unique structure of 2D nanomaterials can provide these materials with properties that cannot be achieved with conventional bulk materials.
- The possibility of assembly into three-dimensional (3D) architectures can also be attributed to the advantages of 2D nanomaterials [17].
- In addition, it should be taken into account that only one or two crystallographic planes are involved in gas-sensitive effects in 2D nanomaterials. This, as in the case of 1D structures, greatly simplifies the modeling of processes occurring on the surface of these materials and contributes to a better understanding of the nature of gas-sensitive effects [5,18]. The same factor can contribute to better sensor selectivity.
- The advantages of 2D structures also include their relatively large lateral size, which, in contrast to 1D nanostructures, greatly facilitates the formation of Ohmic contacts, but, unfortunately, does not completely solve this problem.

At present, based on various 2D nanomaterials, a large number of prototypes of gas sensors with increased sensitivity to various gases have been manufactured. An analysis of these results, as well as methods used to synthesize 2D structures, can be found in numerous reviews devoted to the consideration of these materials [16,19,20,21,22,23,24,25,26,27,28,29,30,31,32]. Therefore, these topics will not be considered in this article.

## 3. Limitations Arising from the Development of Gas Sensors Based on 2D Nanomaterials

The results of testing gas sensors based on 2D nanomaterials showed that, along with the undeniable advantages of these materials, the following difficulties might arise when using 2D nanomaterials to develop high-sensitivity gas sensors [33,34,35,36]:

First, it is very difficult to grow 2D nanomaterials that are large but thin using traditional approaches to metal oxide synthesis. When materials take the form of a real 2D nanomaterial, their thickness turns out to be more than 30–50 nm, which does not contribute to achieving the high sensitivity of sensors. To achieve a high-sensor response, the thickness of the 2D nanomaterial should be comparable with the Debye length (2–5 nm) [37]. Only in this case, modulation of the surface space charge region due to a change in the surface charge caused by adsorption–desorption and catalytic processes can have a noticeable effect on the conductivity of the gas-sensitive layer. However, if we try to synthesize nanosheets with a smaller thickness using traditional approaches, then, in the end, we will get a material that differs little from conventional nanocrystals used in conventional gas sensors [37].

**Figure 1:**
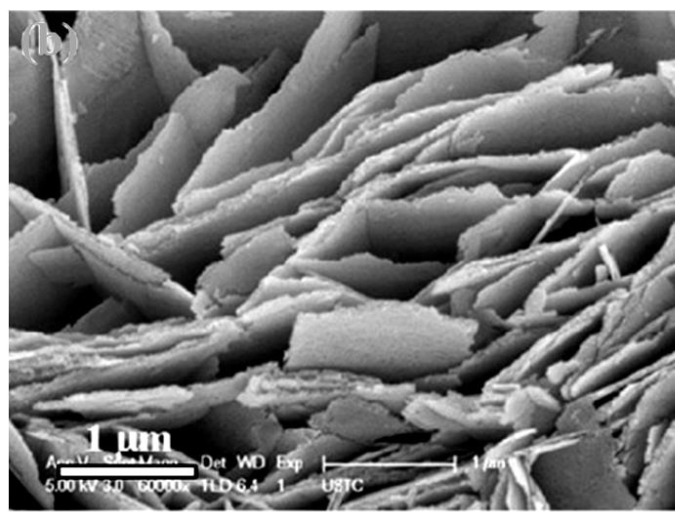
Field-emission-scanning electron microscopy (FE-SEM) image of the ZnO nanosheets. Reprinted with permission from [39]. Copyright 2012: Elsevier.

**Figure 2:**
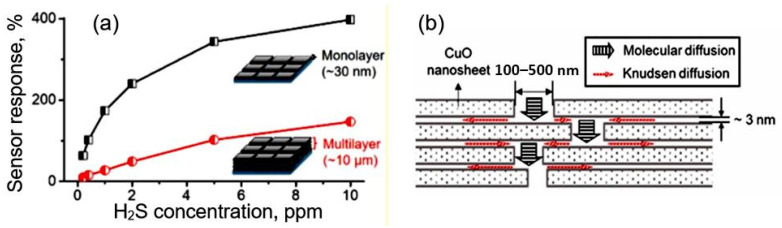
(**a**) Sensor response of monolayer- and multilayer-based sensors to H_2_S; (**b**) Model of a multilayer film with lamellar structure. Reproduced from [38]. Published 2019 by ACS as open access.

If 2D nanomaterials are in the form of large plates, then, in this case, when using thick-film technology for forming a gas-sensitive layer, there is also a threat of forming a gas-sensitive material with a denser arrangement of nanosheets (see Figure 1). This makes it difficult for gas to penetrate the inter-sheet space, further reducing sensitivity and significantly increasing the response and recovery time. This statement has been experimentally confirmed by Miao et al. [38], who established that an increase in the number of nanosheets in gas-sensitive layers, as previously predicted, is accompanied by a decrease in sensitivity (see Figure 2) and an increase in the response time.

**Figure 3:**
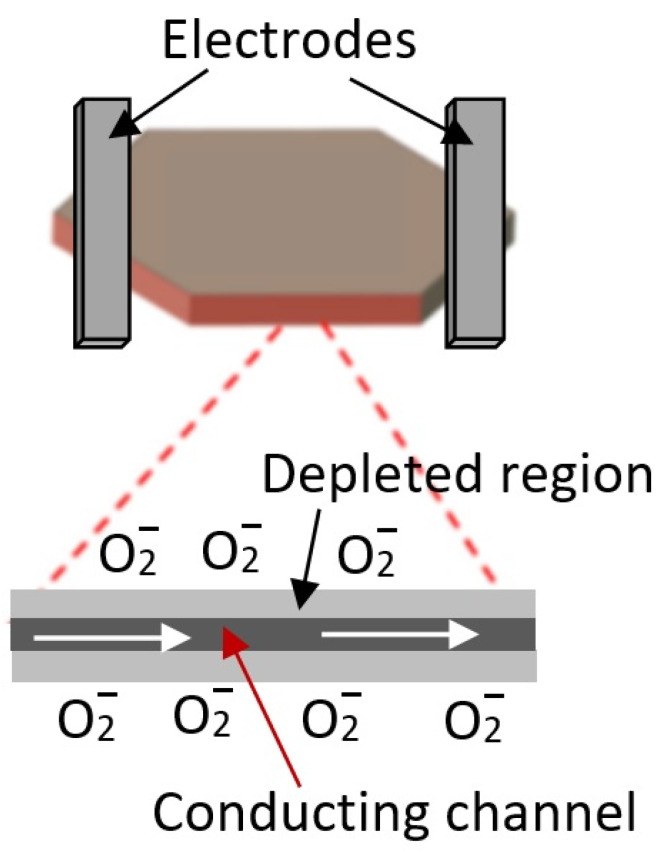
Schematic diagram of gas sensor based on individual 2D nanosheets.

Of course, it is possible to fabricate gas sensors based on individual nanosheets (Figure 3). In such structures, the sensor response is determined either by the resistance of metal–metal oxide contacts or by the conductivity of the nanosheet itself, which is modulated by a change in the width of the space-charge region upon interaction with the gas environment. In this case, the sensor response kinetics is not controlled by diffusion processes. In this case, technological difficulties arise in the manufacture of sensors, especially when organizing mass production, described in [5]. These include separating, manipulating, and fixing nanosheets in the right place on the sensor platform, as well as manufacturing low-resistance ohmic contacts.

Some of the problems associated with the excessive thickness of nanosheets have been solved on the basis of new technologies [22,40] using hydro/solvothermal synthesis [41,42], the interface-mediated synthesis method [43], 2D-oriented attachment method [44], 2D-templated synthesis [45], self-assembly of nanocrystals [46], and the on-surface synthesis method [47].

Using this strategy, 2D structures of TiO_2_, ZnO, Co_3_O_4_, WO_3_, Fe_3_O_4_, V_2_O_5,_ and MnO_2_ nanosheets with planar sizes from 0.2 μm to 10 μm and thicknesses ranging from 1.6 nm to 5.2 nm (corresponding to 2−7 stacking monolayers) have been prepared [48,49]. It is important to note that metal oxides of this thickness must have unique properties in terms of gas-sensing effects. For example, five-atomic-layer-thick 0.66 nm SnO_2_ nanosheets have 40% surface atom occupancy by highly-reactive surface Sn and O atoms with low coordination numbers [49]. These surface atoms could serve as adsorption centers to efficiently adsorb gaseous molecules and/or catalytically active sites, favoring the surface reactions of adsorbed species.

However, it turned out that the smaller the thickness of the nanosheets, the worse their mechanical strength and stability. Wang et al. [50] drew attention to this in their research. By studying the synthesis process of ~10 nm thick WO_3_ nanosheets and analyzing the parameters of gas sensors, they found that the stability of much thinner WO_3_ nanosheets is worse, and the removal of P123 templates was accompanied by the formation of large agglomerates, which led to a decrease in sensor response. Zhang et al. [51] also pointed out the importance of this process. They noted that sensors with highly agglomerated ZnO nanosheets have significantly poorer gas-detection performance. This is natural since the presence of free channels for the penetration of gases and an open space between the nanosheets is a prerequisite for good gas permeability of the sensitive layer, which is necessary to achieve high sensitivity and fast response [37].

Studies have shown that several approaches can be used to increase the gas permeability of the sensitive layer based on two-dimensional structures. For example, Cao et al. [52] used the surface decoration of In_2_O_3_ nanosheets with WO_3_ nanoparticles for this purpose. As a result, both an increase in sensitivity and an improvement in the kinetics of the sensor response were achieved. It can be assumed that the WO_3_ nanoparticles provided the required gap between the In_2_O_3_ nanosheets. The same approach was taken by Yin et al. [53], who showed that the response of sensors with WO_3_ nanosheets coated with SnO_2_ nanoparticles to 50 ppm acetone vapor was 10 times higher than that of sensors based on pristine WO_3_ nanosheets. However, it seems that a more promising approach is the use of porous 2D structures, the synthesis technology of which was considered in the previous sections.

## 4. Advantages of Porous 2D Nanostructures for Application in Gas Sensors

An analysis of the results obtained allows us to highlight the following advantages of porous 2D nanostructures for the development of gas sensors. At first, porous 2D materials can combine the structural advantages of both 2D and porous materials and thus exhibit outstanding chemical and physical properties [10]. Due to the porosity of the 2D materials, more boundaries and edges can be introduced, and nearly all the framework of porous 2D materials can be exposed to the surrounding gas. This means that more accessible surface area and active sites, such as steps, edges, kinks, terraces, and corner atoms with low-coordinated binding states, will be available for interaction with gas surroundings [37]. For example, it is reported that in porous Co_3_O_4_ nanosheets, the presence of pores decreased the coordination number of Co^3+^ atoms to unsaturated four or even three, which served as active sites for many oxidation reactions [54]. In addition, these numerous high-energy sites on the surfaces of metal oxide NFs and NSs can serve as centers for nucleation and adsorption of noble metals and other materials used to functionalize the surface and increase its catalytic activity [55,56]. Similar conclusions were made in [57] based on an analysis of the difference in gas-sensing performance between hole-rich WO_3_ and non-porous WO_3_ nanosheets. They explained the improved response of the sensor based on porous WO_3_ nanosheets to C_2_H_6_S_3_ by the participation of active-edge centers on hole-rich WO_3_ nanosheets in the gas-sensing effect. DFT calculations confirmed that the edge regions show higher adsorption energy and greater charge transfer for gas, and the W–S bond is formed at the edge regions due to strong chemical adsorption.

Second, pores improve the gas permeability of the formed gas-sensitive layer [37]. This means that both the access of gas molecules to the inner surface of the gas sensing layer and the mass transfer of reaction products are facilitated, which increases the efficiency of reactions occurring on the surface of metal oxides [12,37,58,59]. As is known, the mass transfer process is one of the decisive processes determining the performance of any chemical reaction.

**Figure 4:**
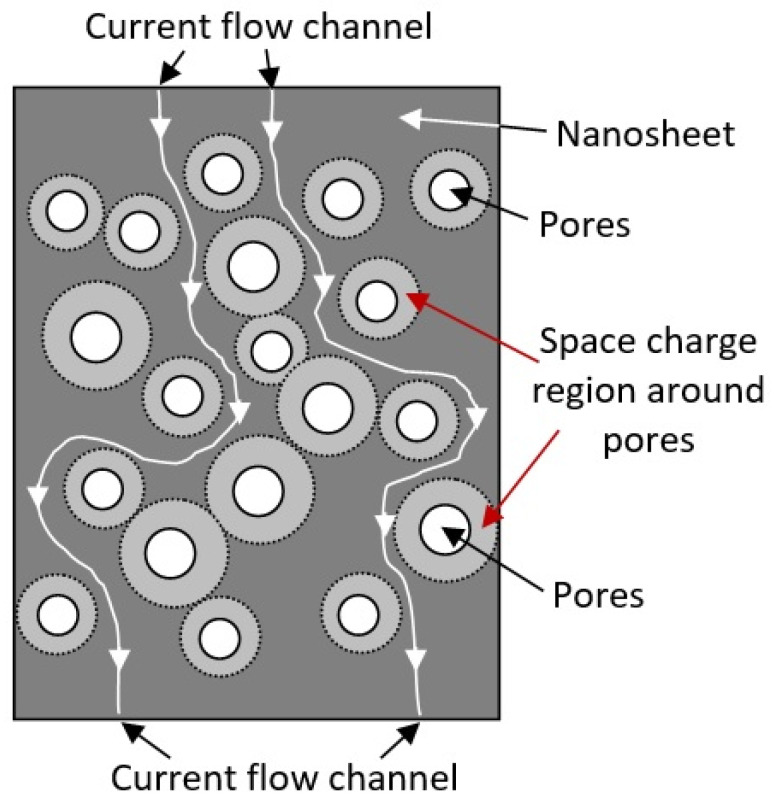
Diagram illustrating the mechanism of current flow channel formation in porous two-dimensional nanostructures. When oxidizing gases are detected, the space charge regions expand, and current flow channels are blocked.

Third, the appearance of pores in nanoflakes and nanosheets makes it possible to form a conductive network in them, which enhances the influence of the atmosphere on the resistance of 2D nanoflakes and nanosheets. This effect is associated with the formation of space charge regions around the pores, which, when the gas atmosphere changes, can also participate in the modulation of the conductivity of nanoflakes and nanosheets (see Figure 4). In this case, the maximum effect should be achieved at a distance between the pores comparable to the Debye length. This makes it possible to block the conduction channels between the pores even at nanosheet thicknesses significantly exceeding the Debye length. A similar effect is often used to explain the increased sensitivity of gas sensors based on porous metal oxide [12,37,58,60,61]. This means that high sensitivity of the sensors can be achieved even with a large thickness of nanoflakes and nanosheets, which cannot be obtained using monolithic (nonporous) nanoflakes and nanosheets. For example, Duy et al. [41], Chen et al. [62], and Choi et al. [63] have shown that gas sensors based on porous 2D nanostructures can indeed have high sensitivity even at nanosheet thicknesses of 80–100 nm.

At the same time, it can be assumed that with an equal volume of pores in nanosheets, a large number of small pores is preferable for gas-sensor development, compared to a small number of large pores. The large thickness of the nanoflakes, in turn, should also improve the stability of the parameters of sensors based on them. This approach has been intensively developed in recent years. The parameters of gas sensors using porous 2D structures are given in Table S1 (Supplementary Materials). Based on the foregoing, it can be argued that the use of porous 2D materials in developing solid-state gas sensors is indeed a promising direction that can significantly increase the sensitivity and improve the rate of response of gas sensors. Of course, to achieve such a result, a corresponding optimization of the electrophysical properties and structure of 2D nanomaterials is necessary.

## 5. Features of Manufacturing Porous 2D Structures

### 5.1. Creation of Nanoholes in 2D Structures

Currently, there are two main approaches to fabricating porous 2D nanostructures: nanoflakes and nanosheets. The first approach combines methods based on creating nanoholes in already-formed 2D structures. Obtaining nanoholes in 2D structures, in this case, is carried out as a result of various post-processing treatments:

#### 5.1.1. Reorganization of the Structure or Phase Transition

The most common is the group of methods that result in the reorganization of the structure of 2D nanomaterials or phase transition. The experiment has shown that the reorganization of the structure of a 2D material can be observed when the oxidation states of atoms that form 2D structures change or when some cations or anions from the composition of 2D nanomaterials are replaced by other cations and anions [64,65,66,67]. The first can occur during the oxidation or reduction of atoms forming 2D structures, and the second as a result of ion exchange reactions, which lead to structural-chemical deformation in the grid of chemical bonds and the formation of nanoholes. Studies have shown that changing oxidation states can be implemented for nanoflakes containing transition metals, such as V, Co, Cu, Cr, Fe, etc., with multiple stable oxidation states. For example, such a reaction was used by Hong et al. [67] in the synthesis of CoOOH NSs. In this work, for the synthesis of CoOOH NSs with a large number of holes about 1 nm in size, a simple coordination-oxidative-hydrolysis method was used. The formation of porous CoOOH NSs was facilitated by the Co(II) → Co(III) oxidation reaction occurring in a solution of an oxidation reagent H_2_O_2_.

An example of a reverse reaction is the reduction of HCoO_x_ NSs with a solution of SnCl_2_ at 90 °C, proposed by Jang et al. [68]. As was established, this reaction leads to the reduction of Co(III) cations to the oxidation state of Co(II) and the oxidation of Sn(II) cations to Sn(IV). As a result, nanoholes with a size of 2–50 nm are formed in HCoO_x_ NSs. During heat treatment of metal oxides in an atmosphere of reducing gases, such a mechanism can also be realized. In particular, Li et al. [69] observed the formation of nanoholes with a size of 30–40 nm in CoMnO_4_ nanosheets after thermal treatment in a mixture of hydrogen and argon at 400 °C. Li et al. [69] explain the appearance of nanoholes by the removal of some oxygen atoms from the composition of metal oxide.

**Figure 5:**
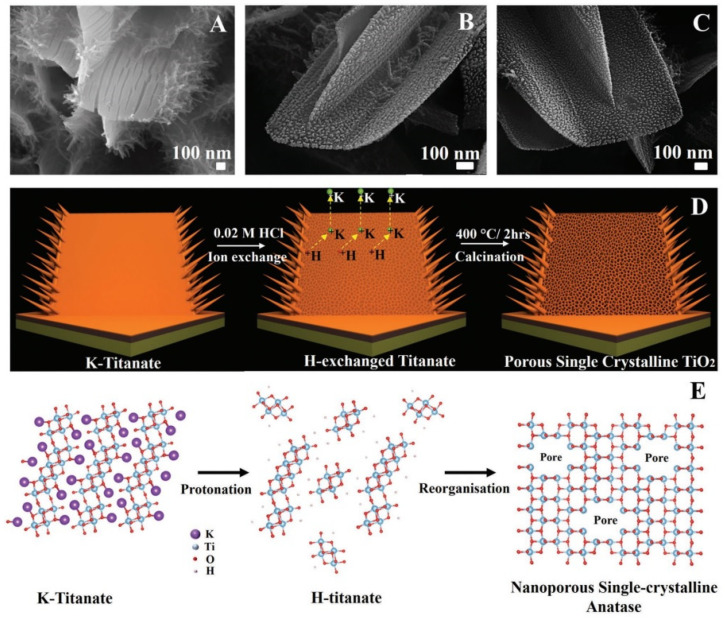
Nanostructure evolution mechanism. SEM images of (**A**) K-titanate, (**B**) H-titanate, and (**C**) porous single-crystalline TiO_2_, respectively. (**D**) Schematic illustration of converting K-titanate to porous single-crystalline TiO_2_ via ion exchange followed by calcination. (**E**) Atomic crystal model of crystal structure changes during ion exchange and calcination processes. Reprinted with permission from Butburee et al. [70]. Copyright 2018: Wiley.

As for the preparation of porous NSs using a cation exchange reaction, a good example of such a method is the process of forming porous TiO_2_ nanosheets based on the treatment of K-titanate films in an HCl solution [70]. As a result of this treatment, the reaction of ion exchange of K^+^ cations to protons and the formation of H-titanate occurs (see Figure 5). After heating the obtained samples in the air at 400 °C, the transformation of its structure and the formation of porous TiO_2_ NSs with the anatase crystal structure and nanoholes of 2–4 nm in size are observed. In some works, the appearance of pores during phase transition is associated with rapid kinetic and deformation stresses caused by a large mismatch between the lattices of two phases present in metal oxides [71,72].

**Figure 6:**
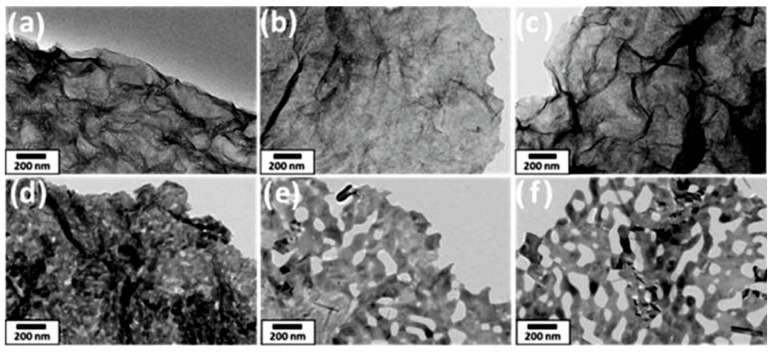
TEM images of MnO_2_ nanosheets after annealing in air at different temperatures: (**a**) as-synthesized, (**b**) T_an_ = 300 °C, (**c**) T_an_ = 400 °C, (**d**) T_an_ = T_an_ = 500 °C, (**e**) T_an_ = 600 °C, (**f**) T_an_ = 700 °C. Reprinted with permission from Adpakpang et al. [73]. Copyright 2018: Wiley.

The experiment has shown that phase transition with pore formation can also occur in the process of simple thermal annealing of metal oxides. For example, Adpakpang et al. [73] have found that calcinating the layered MnO_2_ nanosheets in the ambient atmosphere can result in the formation of holey Mn_2_O_3_ nanosheets with mesopores (see Figure 6). The holes originate from the removal of oxygen ions during the reductive phase transition, and the pore dimensions were directly related to the heating temperature.

#### 5.1.2. Etching of Synthesized 2D Structures

This method works most successfully when processing two-dimensional structures in a non-thermal gas plasma. An example of such treatment is the preparation of porous heterostructural Co_3_O_4_-CoO nanosheets after their treatment in cold Ar-H_2_ plasma [74]. With an increase in the treatment time in the Ar-H_2_ plasma, gradual etching of the Co_3_O_4_-CoO NSs is observed, which leads to the formation of numerous nanoholes. At first glance, the formation of holes occurs in the areas with increased defectiveness, where the etching rate is higher. However, Ar-H_2_ plasma treatment is also accompanied by partial oxide reduction [75], i.e., loss of lattice oxygen and defect generation. This is naturally accompanied by a violation of the symmetry and arrangement of atoms in the NSs and leads to structural changes, which should contribute to the formation of pores.

#### 5.1.3. Chemical Dissolution of Individual Components of 2D Materials

**Figure 7:**
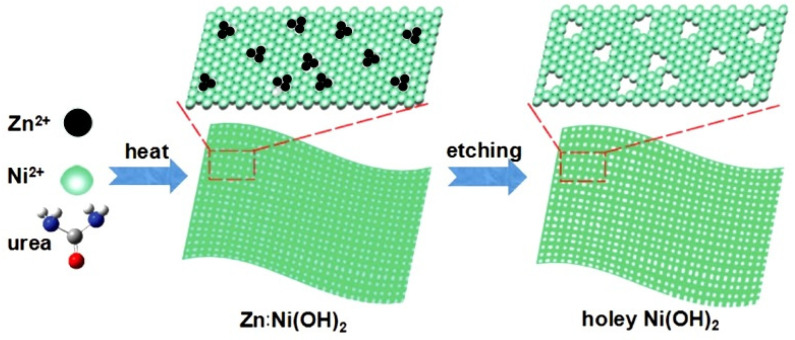
Schematic illustration for the formation of clean and freestanding tiny holey 2D Ni(OH)_2_ nanosheets. Reprinted with permission from Kong et al. [76]. Copyright 2017: Wiley.

This method is based on chemical etching, which makes it possible to remove some of the atoms of a certain type from the compound and form nanoholes due to ongoing structural changes (see Figure 7). The main problem of this method is the selection of appropriate easily-soluble components, their inclusion in metal oxides or oxyhydroxides in which pore formation is necessary, and the creation of conditions for their selective etching. At present, this approach is usually implemented by introducing Zn(II), Al(III), Ga(III), Cr(III), or Na cations into the composition of NSs at the stage of their synthesis, followed by their dissolution in an alkaline medium. An example of the implementation of this method is the process of nanopore formation in Ni(OH)_2_ NSs proposed by Kong et al. [76]. For chemical dissolution of Zn in Ni(OH)_2_:Zn, NSs were treated in 5 M NaOH solution heated to 85 °C for 30 min. As a result, nanoholes up to 5 nm in diameter were formed in such NSs. The same approach was taken by Lu et al. [77] for the formation of holey LiCoO_2_ nanosheets. The formation of pores occurred due to the extraction of lithium from LiCoO_2_ during acid etching.

#### 5.1.4. Removal of Volatile Components

**Figure 8:**
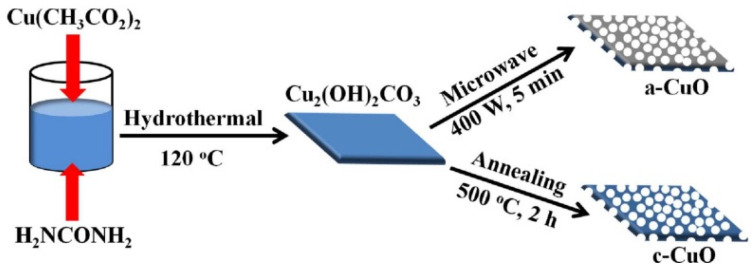
The designed synthetic route of the c-CuO and a-CuO porous nanosheets. Reprinted from Tian et al. [78]. Published 2020 by Wiley as open access.

Removal of volatile components from the composition of 2D structures by thermal heating in a controlled gaseous environment or vacuum is perhaps the simplest and most commonly used method [78,79]. This treatment removes water molecules and other highly-volatile components from NSs, resulting in the formation of nanoholes due to the reformatting of metal–oxygen bonds and phase transition. This means that this method, in principle, can be attributed to one of the varieties of phase transition methods. One example of this approach is shown in Figure 8. As a result of heat treatment of Cu_2_(OH)_2_CO_3_ synthesized by the low-temperature hydrothermal method, a dramatic change in the structure is observed, associated with the decomposition of Cu_2_(OH)_2_CO_3_ (reaction (1)) and accompanied by the formation of highly porous CuO nanosheets with the pore sizes around 15–30 nm [78].

Cu_2_(OH)_2_CO_3_ → 2CuO + H_2_O↑ +CO_2_↑ (1)

The same process occurs during the heat treatment of zinc nitrate hydroxide hydrate (ZNH) polygonal nanoflakes at 300–600 °C [62]. ZNH polygonal nanoflakes were synthesized using a microwave-assisted approach. As a result of heat treatment, uniform porous ZnO polygonal nanoflakes are formed with a thickness of about 50–90 nm, an average size of less than 900 nm, and pore size in the range of 30–60 nm. Cobalt-oxalate coordination complex (CoC_2_O_4_·2H_2_O), thin sheets synthesized by the hydrothermal method after thermal treatment at 400–600 °C, also transforms into porous 2D Co_3_O_4_ thin nanosheets with a pore size of 5–100 nm [80].

#### 5.1.5. Physical Methods

There are also proposals to form pores in 2D materials by physical methods, such as high-energy light, ion, or electron-beam exposure [10]. For example, a focused ion beam (FIB) can provide high-energy heat to ‘‘drill’’ pores of a controllable size. However, it must be admitted that this method is unlikely to find application in the development of sensors for the market.

### 5.2. Direct Synthesis of Porous 2D Nanostructures

**Figure 9:**
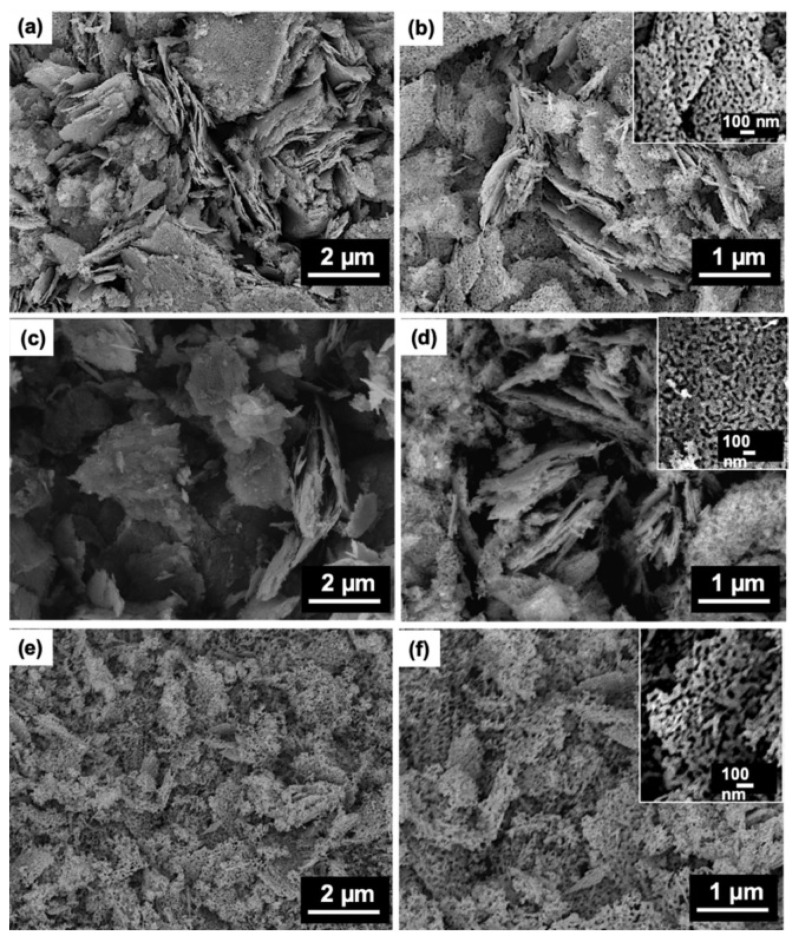
SEM images of ZnO-In_2_O_3_ porous nanosheets of various compositions synthesized by solvothermal method at different magnifications: (**a**,**b**) –Zn^2+^/In^3+^ = 10:0 M ratio; (**c**,**d**) Zn^2+^/In^3+^ = 10:2 M ratio, and (**e**,**f**) Zn^2+^/In^3+^ = 10:6 M ratio. Reprinted with permission from Yan et al. [82]. Copyright 2021: Elsevier.

The second approach to the creation of porous two-dimensional structures is based on the direct synthesis of such structures. Undoubtedly, this is the most optimal approach to fabricating porous 2D structures since it excludes the multi-stage fabrication process. The most common method for the direct synthesis of porous two-dimensional structures is considered to be the hydrothermal/solvothermal method due to its high versatility, high yield, and high efficiency. The hydrothermal/solvothermal method can carefully control the crystal structure and morphology of synthesized materials, which cannot be achieved at atmospheric pressure. For example, porous ZnO [79] and Ni-Co bimetal oxide nanosheets [81] have been synthesized using this method. The one-pot solvothermal method was used by Yan et al. [82] for the synthesis of segregated ZnO-In_2_O_3_ porous nanosheets. SEM images of these nanosheets are shown in Figure 9. However, large aggregation is observed in ZnO nanosheets (Figure 9a,b). With an increase in the In_2_O_3_ content, aggregation decreases (Figure 9c,d), and in samples with the highest In_2_O_3_ content, aggregation is not observed on SEM images (Figure 9e,f). This suggests that the addition of indium ions suppresses the stacking of the nanosheets, which results in high dispersion and efficient gas diffusion inside the ZnO-In_2_O_3_ nanosheets. According to Yan et al. [82], the porous structure of the nanosheets could be attributed to the loss of volatile gas, such as H_2_O and CO_2,_ during the synthesis procedure. Zhu et al. [83] reported the synthesis of a 3D hierarchical Co_3_O_4_ structure assembled by ultrathin nanosheets with a pore size of 8–10 nm via direct hydrothermal decomposition of an aqueous solution of cobalt nitrate in the presence of benzoic acid (BA). In addition to binary metal oxides, ternary materials with porous 2D structures, such as porous ultrathin BiWO_6_ nanosheets [84], have also been synthesized by the hydrothermal method. Although the hydrothermal method is an effective strategy for the fabrication of porous 2D materials, continuous efforts should still be made to investigate the corresponding working mechanism of hydrothermal treatment as well as the influence of processing conditions on the microstructure and morphology of porous 2D materials for precise control. For example, Li et al. [85] have found that during the hydrothermal synthesis of SnO_2_ flowerlike nanostructures, the semi-blooming nanoflowers assembled by high-yield and uniform-ordered mesoporous nanosheets are formed after calcination only when the reaction time is extended to 12 h. When the hydrothermal time is less than 12 h, the product shows a prototype of flowerlike architectures comprised of relatively uniform nanosheets. If the hydrothermal time increases to 16 h, flowerlike hierarchical nanostructures are destroyed into irregular nanosheets.

In addition, it should be kept in mind that, in most cases, the formation of porous metal oxide 2D nanostructures requires annealing at temperatures of 300–500 °C at the final stage to transfer hydroxyls to the metal oxide phase [79,81,85,86]. For example, Ryu et al. [87] synthesized ZnO nanosheets by the hydrothermal method and found that the nanosheets became highly porous only after calcination at 350 °C for 5 h. This means that the formation of pores in such structures often occurs according to the mechanism corresponding to the phase-transition method. For example, Sharma et al. [86] showed that the formation of porous NiMoO_4_ nanosheets requires annealing at 400 °C in the presence of air, during which there is a transition from NiMoO_4_·xH_2_O, which has a 3D honeycomb structure, to NiMoO_4_ porous nanosheets, which have 3D rectangular structures. When using other synthetic methods, such as sol-gel [88], electrodeposition [89], chemical bath deposition [90], and wet chemical method [91], designed for the synthesis of ZnO, WO_3_, SnO_2_, Co_3_O_4_, In_2_O_3_, NiMoO_4_, CeO_2,_ and other metal oxide-based porous 2D structures, post-processing high-temperature annealing is also generally required.

**Figure 10:**
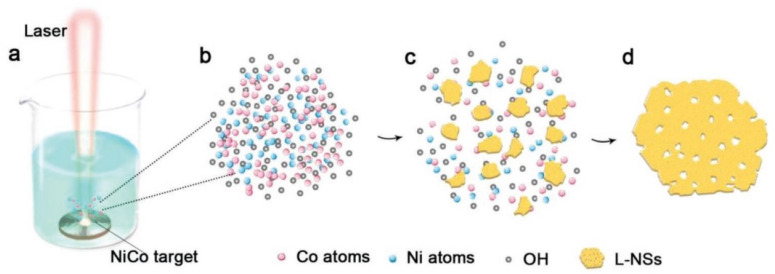
The formation of L-NSs. (**a**) Laser ablation of CoNi alloy target in 1 m KOH solution, (**b**) mixed vapor of cobalt, nickel, and OH^−^, (**c**) formation of hydroxide debris in the vapor, (**d**) formation of L-NSs via orientation attachment. Reprinted with permission from Wang et al. [93]. Copyright 2019: Wiley.

Of course, there are other methods for the direct synthesis of porous 2D materials [92,93,94]. For example, Wang et al. [93] synthesized Co_0.75_Ni_0.25_(OH)_2_ nanosheets with numerous nanopores by pulsed laser ablation in liquid (see Figure 10). Pulsed laser ablation can also be used to form nanocomposites, such as ZnO NSs-CNT [87]. Such hybrids can be promising for the formation of NSs-based gas-sensitive layers with increased gas permeability. CNTs prevent nanosheets from agglomerating.

Zhu et al. [94] suggested using direct calcination of an Sn-based metal-organic framework that was prepared via a facile wet chemistry method with deionized water as solvent at 80 °C to form porous SnO_2_ nanosheets intended for fabricating highly-sensitive formaldehyde sensors. The porous SnO_2_ nanosheets are found to be constructed by the interconnection of ultrafine nanoparticles with sizes of 4–9 nm, comparable to the Debye length of SnO_2_. The synthesized SnO_2_ nanosheets demonstrated a relatively narrow pore-size distribution of 2–12 nm, with a peak occurring at 7 nm.

**Figure 11:**
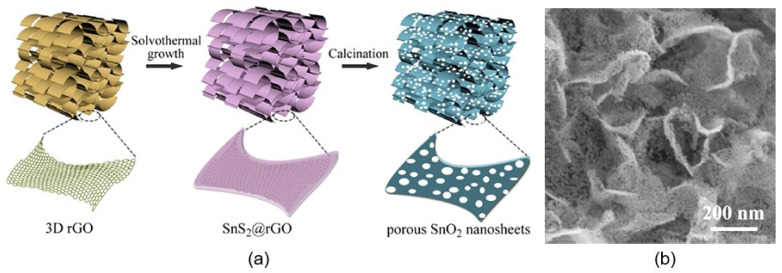
(**a**) Schematic diagram of the synthesis procedure and **(b**) SEM image of porous SnO_2_ nanosheets. Reprinted with permission from Huang et al. [97]. Copyright 2022: Elsevier.

There are also attempts to form porous 2D structures using template methods [72,95,96]. For example, porous 2D metal oxides can be obtained by depositing metal oxides on a porous hard template and then removing it. As a hard-templating material, porous silicon or Al_2_O_3_ can be used, the porosity of which will also determine the porosity of the formed 2D metal oxide. However, this method is laboratory in nature and is unacceptable for large-scale application. Huang et al. [97] have taken a different approach to preparing porous 2D nanostructures. Cross-linked porous tin dioxide nanosheets were obtained using three-dimensional reduced-graphene oxide as a hard template followed by an annealing process at 550 °C in the air for 4 h (Figure 11). During this annealing, SnS_2_@rGO precursor is transformed in semiconductor metal oxide nanostructures with large specific surface areas (77.4 m^2^/g), high porosity, and, as a result, improved gas-sensing characteristics. The pore size was in the range of 2–28 nm.

**Figure 12:**
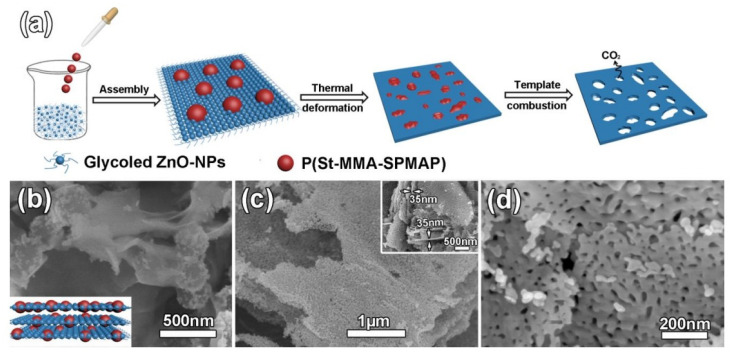
(**a**) Schematic illustration of the ZnO-MSN synthesis, (**b**) SEM images of the layer assembled ZnO/P(St-MMA-SPMAP) composites with the inset showing the perspective view of schematic image, (**c**) low magnification SEM image with the inset showing the thickness of ZnO-MSN and (**d**) high magnification SEM image of ZnO-MSN. Reprinted with permission from Liu et al. [98]. Copyright 2016: Elsevier.

As for the soft-template method, it is more suitable for practical use. The soft-template method has distinct advantages in preparing porous-structured 2D nanomaterials because it is flexible for controlling the morphology of prepared materials. In particular, ZnO mesoporous single-crystal nanosheets (ZnO-MSN) with exposed {0001} polar facets and pore size ~35 nm were synthesized using this method [98]. Soft templates were 250 nm monodispersed colloids of poly(styrene-methyl methacrylate-sulfo propyl methacrylate potassium) (P(St-MMA-SPMAP)) (see Figure 12).

## 6. Performances of Gas Sensors Based on Porous 2D Nanostructures

As shown in Table S1, porous nanosheets and nanoflakes of various materials, from binary metal oxides to complex compounds, such as LaFe_1-x_P_x_O_3-δ_ [99], have been tested for the manufacture of gas sensors. However, the main 2D materials used to manufacture gas sensors are still metal oxides such as ZnO [39,100,101], WO_3_ [90], In_2_O_3_ [102,103], SnO_2_ [88,104], CuO [105], Co_3_O_4_ [106], NiO [107], CeO_2_ [108], LaFeO_3_ [55], and TiO_2_ [56] which have physical—chemical properties most suitable for work in aggressive environments [15]. As a rule, gas sensors were fabricated using conventional ceramic and thick-film technology [13]. The maximum sensitivity of the developed sensors, depending on the material used, its structural parameters, and the nature of the detected gas, is observed in the temperature range from RT [102] to 400 °C [41,109]. The operation temperature depends on the semiconductor used and its structural condition. However, as a rule for most sensors, the maximum sensory response to reducing gases is observed at temperatures of 200–340 °C [88,97,110], and to oxidizing gases, at a temperature of 100–200 °C [62,90]. This difference is associated with a different mechanism of sensory response to these gases and is typical of all metal oxide sensors [15,111,112].

**Figure 13:**
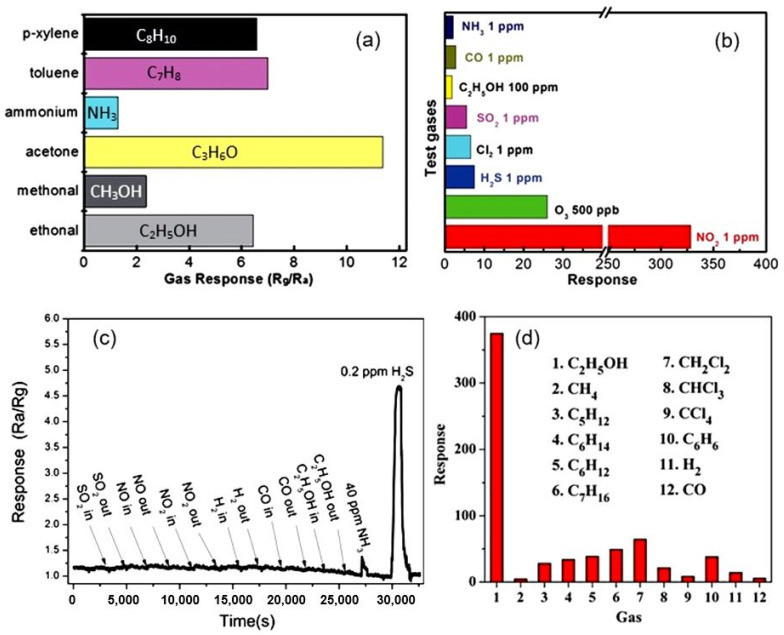
(**a**) The conductometric responses of sensors based on porous 2D Co_3_O_4_ nanosheets to several reducing gases with concentrations of 100 ppm at 150 °C. Reprinted with permission from [106]. Copyright 2015: RSC; (**b**) Cross-responses of the sensor based on 2D porous In_2_O_3_ nanoflakes to various test gases at 140 °C. Reprinted with permission from [116]. Copyright 2015: RSC; (**c**) Response and recovery curves of the porous CuO nanosheet-based gas sensors to difference gases (SO_2_, NO, NO_2_, H_2_, CO, C_2_H_5_OH, NH_3_) of 40 ppm and 0.2 ppm H_2_S at room temperature. Reprinted with permission from [105]. Copyright 2016: ACS; (**d**) The cross-response of the ZnO-based sensor to ethanol and other 11 interfering gases at 400 °C. Reprinted with permission from [39]. Copyright 2012: Elsevier.

The best examples of gas sensors developed on the basis of porous 2D structures also have a fast response and an increased sensitivity to various gases that exhibit both reducing (CO, H_2_S, NH_3_) [113,114] and oxidizing properties (O_3_, NO_2_) [115,116]. High sensitivity is also shown to the vapors of organic solvents [51,101,117]. Although metal oxide sensors are not selective-gas sensors (see Figure 13a), under certain conditions, sensors based on 2D materials, as well as conventional metal oxide gas sensors, can exhibit good selectivity to gases (Figure 13b,c), such as O_3_, NO_2_, H_2_S, which have specific properties [15,118,119]. Zhang et al. [39] reported that their ZnO-based sensors developed using porous 2D nanostructures had high selectivity to ethanol (Figure 13d). However, the mechanism of such selectivity is unclear since similar ZnO-based sensors developed in other laboratories did not have such a pronounced selectivity to ethanol [19,62,120]. This suggests that the technology for the synthesis of nanosheets and nanoflakes plays an important role in the formation of their gas-sensitive properties, and this role needs to be studied.

**Figure 14:**
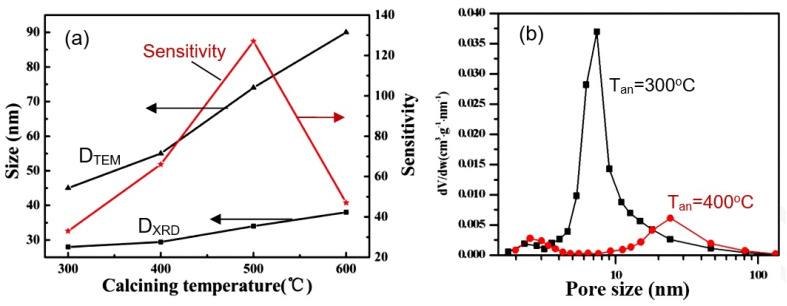
(**a**) Effect of annealing on crystallite size (D_TEM_ and D_XRD_) and sensitivity of sensors based on porous ZnO nanoflakes. Reprinted with permission from [62]. Copyright 2011: RSC; (**b**) Pore−size-distribution curves of 3D hierarchical ZnO porous structure after annealing at 300 °C and 400 °C. Reprinted with permission from [120]. Copyright 2015: Elsevier.

The important role of technology in the synthesis of 2D structures used to develop gas sensors is evidenced by the data presented in Table S1. They show that even when using the same synthesis method, depending on the used precursors, solvents, temperature, and time regimes of synthesis, the parameters of sensors can differ radically. Studies performed by Chen et al. [62] and Fan et al. [120] showed that in addition to the synthesis conditions, in order to achieve the best parameters of gas sensors, it is also necessary to control the temperature regimes of annealing of already-synthesized 2D structures (see Figure 14a). This is quite understandable since such heat treatment is accompanied by a change in the morphology and size of the crystallites that form nanoflakes or nanosheets (Figure 14a) and the size of the pores formed in them (Figure 14b).

However, as follows from the results presented in [121], it is not always clear what determines the change in sensor parameters—a change in the size and density of pores or a change in the electrical and surface properties of metal oxides under the influence of heat treatment. In particular, Feng et al. [121] found that the maximum response of sensors based on porous NiO nanosheets to N_2_H_4_ at 92 °C was observed after annealing at 700 °C. At the same time, if at a temperature of 500 °C the nanosheets had a mesoporous structure with a large number of pores, then, as the calcination temperature increased to 600 °C, the pore size on the surface of the NiO nanosheets became larger. When the calcination temperature reached 700 °C, the pore size became much larger, and only a few large pores remained on the surface of the nanosheets. Upon reaching the calcination temperature of 800 °C, the NiO nanosheets melted and were destroyed. The described behavior of the nanosheet morphology indicates that the observed change in the parameters of sensors based on NiO NSs during annealing is more consistent with the effect of heat treatment on the bulk and surface properties of NiO.

**Figure 15:**
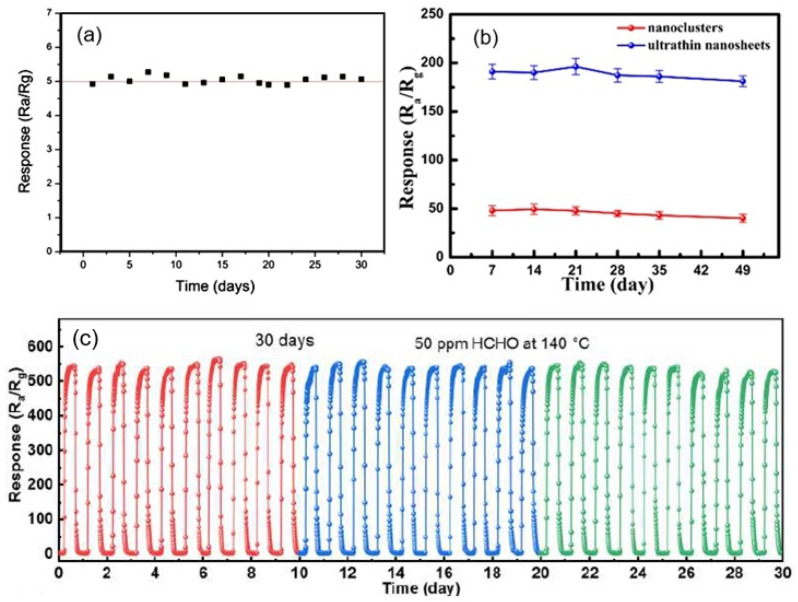
(**a**) Long-term stability of the porous CuO nanosheet-based gas sensor exposed to 200 ppb H_2_S gas at room temperature. Reprinted with permission from [40]. Copyright 2016: ACS; (**b**) Long-term stability of the response of ZnO-based sensor toward acetylacetone at 340 °C. Reprinted with permission from [100]. Copyright 2019: ACS. (**c**) Durability of SnO_2_ porous nanosheet-based sensor of formaldehyde (HCHO). Reprinted with permission from Zhu et al. [94]. Copyright 2021: Elsevier.

Measurements performed in [40,94,100] showed that sensors based on porous 2D nanostructures, as well as conventional metal oxide-based gas sensors, with appropriate fabrication optimization [122,123], are characterized by good temporal stability of parameters (see Figure 15). All this indicates that porous 2D nanostructures are indeed promising materials for the development of gas sensors for various applications.

As for the effect of air humidity on the parameters of sensors based on porous two-dimensional nanomaterials, the results presented in various articles [92,94,124,125,126] show that, similar to conventional metal oxide gas sensors, they are affected by humidity. This effect depends on the metal oxide used, the type of gas detected, the operating temperature, and the side effects. In particular, the mesoporous structure contributes to the condensation of water vapor in nanopores even at a sufficiently low humidity level. Production technology is also of great importance. For example, Spagnoli et al. [124] showed that WO_3_ NSs have less effect on film conductivity, while according to Liu et al. [125], WO_3_ NS-based sensors showed a noticeable effect of humidity on sensor performance. The influence of humidity on the properties of metal oxides and sensors based on them is considered in sufficient detail in [127,128].

**Figure 16:**
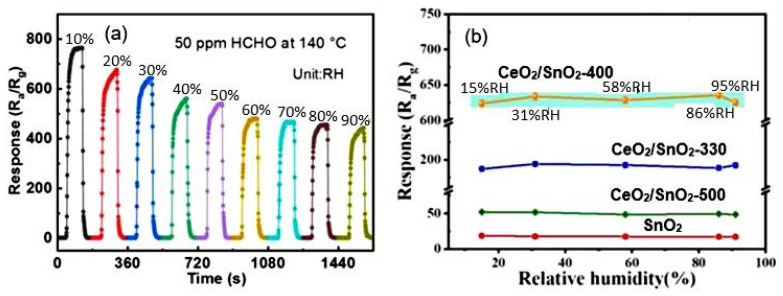
(**a**) Response-recovery curves of SnO_2_ porous nanosheet-based sensor to HCHO under different RH. Zhu et al. [94]. Copyright 2021. Elsevier. (**b**) Relationship between relative humidity and response of porous CeO_2_-SnO_2_ nanosheet-based sensor towards 3-hydroxy-2-butanone, a biomarker for pathogenic bacterium Listeria monocytogenes: 330, 400, and 500 correspond to the annealing temperature. Reprinted with permission from Yang et al. [92]. Copyright 2022: Elsevier.

The typical effect of humidity on the conductometric response of various 2D-based sensors is shown in Figure 16a. It can be seen that the influence of humidity can be significant, and this influence cannot be neglected if the sensor is designed to measure gas concentration. However, the experiment showed that with appropriate calibration and independent measurement of the air humidity, the effect of humidity could be compensated by the electronic method [123]. If the structure and composition of the gas-sensitive material are optimized to reduce the influence of humidity (Figure 16b), and the sensor is used in alarm systems, then, with sufficient sensitivity of the sensors to the detected gas, the influence of humidity can be neglected. This is exactly the situation we have with the CeO_2_/SnO_2_ sensors developed by Zhu et al. [94]. It is believed [94] that the humidity-independent property of CeO_2_/SnO_2_ sensors is mainly attributed to the presence of cerium oxide. CeO_2_ nanoparticles can be considered an absorber of hydroxyl groups due to a facile redox reaction (Ce^3+^/Ce^4+^) [129,130].

**Figure 17:**
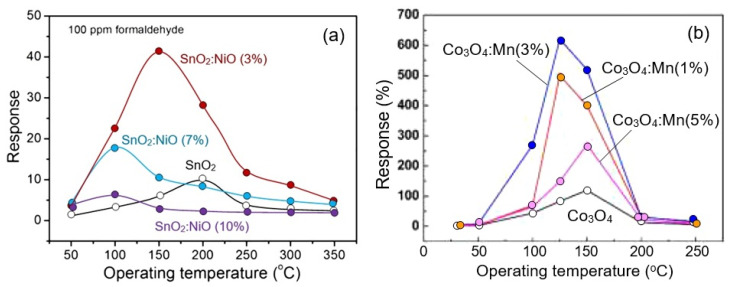
(**a**) Response versus operating temperature of gas sensors based on Pd-modified porous ZnO nanosheets to different gases; (**b**) Response of Pd/ZnO and ZnO-based sensors to different gases at 220 °C. Reprinted with permission from [143]. Copyright 2012: Elsevier.

**Figure 18:**
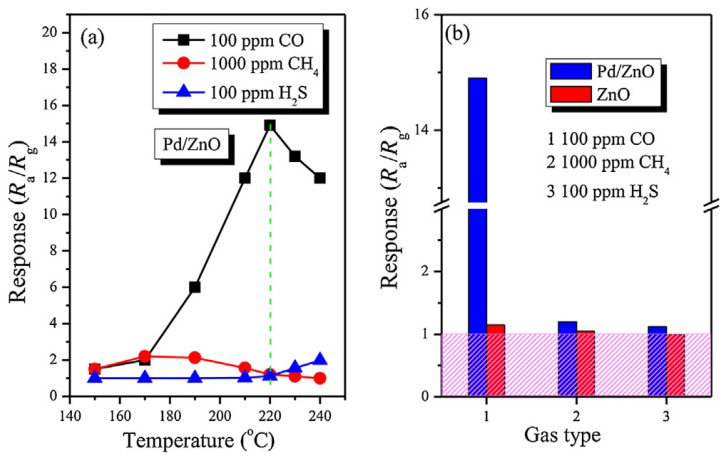
(**a**) Response of sensors based on SnO_2_ and SnO_2_ doped with NiO nanoflowers to 100 ppm formaldehyde. Adapted with permission from [136]. Copyright 2018: Elsevier. (**b**) The responses of bare Co_3_O_4_ and Co_3_O_4_ nanosheets doped with Mn to 100 ppm CO at different temperatures. Reprinted with permission from Qin et al. [133]. Copyright 2022: Elsevier.

It is important to note that, in the fabrication of sensors based on porous 2D nanomaterials, all approaches developed earlier for optimizing the parameters of conventional metal-oxide gas sensors can be used: namely, doping of metal oxides or surface modification with catalytically-active noble metals [56,91,131], metal oxides [52,53,132], II-VI compounds [126], and even phosphorus [133]. Just as in the case of conventional metal oxides [119,134], these approaches provide a significant improvement in sensor performances, such as sensitivity, selectivity, and response and recovery times. For example, Figure 17 shows the influence of surface modification with Pd on the conductometric response of sensors based on 2D porous ZnO nanosheets. It is seen that surface modification strongly increases response and improves selectivity to CO. At the same time, it should be borne in mind that the achievement of the desired result is possible only at a certain concentration of dopants, specific for each metal oxide and doping element, and usually does not exceed a few percent [126,135,136]. Deviation from this value is accompanied by a drop in sensor response (see Figure 18). Moreover, such behavior is observed both during surface modification and bulk doping with noble metals [55,56] and transition metals [133,136]. For example, according to Wang et al. [56], the maximum response of a porous TiO_2_ nanosheet-based sensor to H_2_ was observed when the amount of Pd loaded during impregnation was 1.5 wt%. Even with phosphorus doping of the LaFe_1-x_P_x_O_3-δ_ compound, to achieve the maximum sensory response, the phosphorus concentration should not exceed 1% [99]. The mechanism of such influence is described in [119,134,137,138,139]. If, however, we are applying the second gas-sensitive oxide to the surface of the already formed porous metal-oxide nanosheets, as was done in [140], then completely different patterns can be observed here. For example, in gas sensors based on the SnO_2_ nanorods/2D NiO porous nanosheet composite, the maximum sensory response was observed at a Ni:Sn ratio of 1:0.5 [140]. When developing sensors based on a composite of mesoporous ZnO/Co_3_O_4_ nanosheets, the optimal Zn:Co atomic ratio is 1:0.15 [141].

Porous nanoflakes can also be used to make hybrid nanocomposites. For example, Bai et al. [142] suggested using CuO nanoflakes/rGO nanosheet composites for the detection of NO_2_. The composite was synthesized via an ultra-low-cost hydrothermal process with a thermal treatment. The gas sensor based on the as-synthesized CuO/rGO was fabricated by the spin-coated method and applied to detect NO_2_ gas at room temperature (23 °C). The sensor exhibited a conductometric response of approximately 40 towards 5 ppm NO_2,_ and the limit of detection was down to 50 ppb.

## 7. 3D nanostructures Based on Porous 2D Nanosheets and Nanoflakes

**Figure 19:**
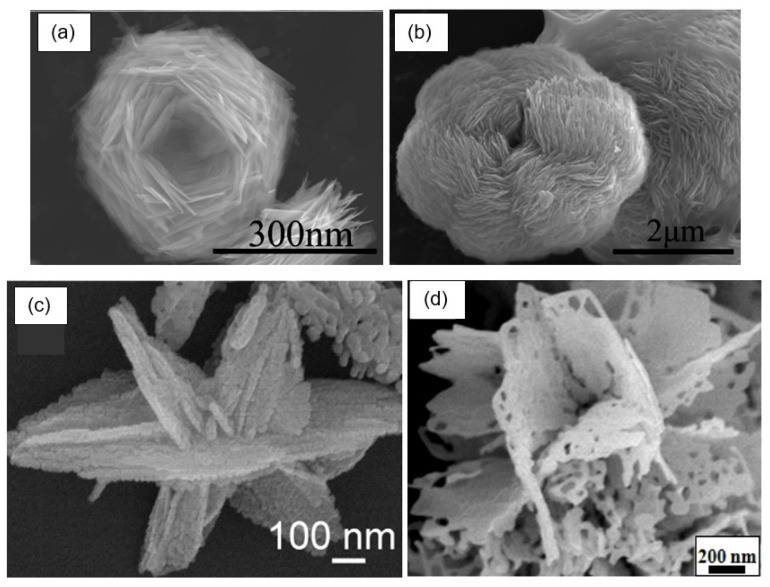
Varieties of 3D structures: (**a**,**b**) SEM images of nestlike 3D ZnO porous structures synthesized at different temperatures. Reprinted with permission from [145]. Copyright 2012: ACS; (**c**) FE-SEM images of In_2_O_3_ hierarchical nanostructure. Reprinted with permission from [102]. Copyright 2012: Elsevier; (**d**) SEM image of porous flowerlike SnO_2_ nanostructures. Reprinted with permission from [146]. Copyright 2015: Elsevier.

The next step in optimizing the parameters of gas sensors based on 2D porous nanoflakes and nanosheets is the development of 3D structures based on them. 3D structures can have a wide variety of shapes: nanoflowers and various hierarchical structures (see Figure 19). It should be noted that the structure of the gas-sensitive layer formed in this way favorably differs from the structure of the gas-sensitive layer formed using traditional thick-film technology [37,144]. If mesoporous or microporous structures are formed as a result of using traditional gas-sensor technology, then 3D structures formed by aggregation of 2D nanosheets and nanoflakes are macroporous. Such structures are optimal for gas sensors since a nanoscale network system formed provides maximum porosity and free gas access to almost all elements of the gas-sensing layer, which facilitates fast and effective gas adsorption onto the entire sensing surface, thereby providing a significant improvement in sensitivity and a reduction in response time.

**Figure 20:**
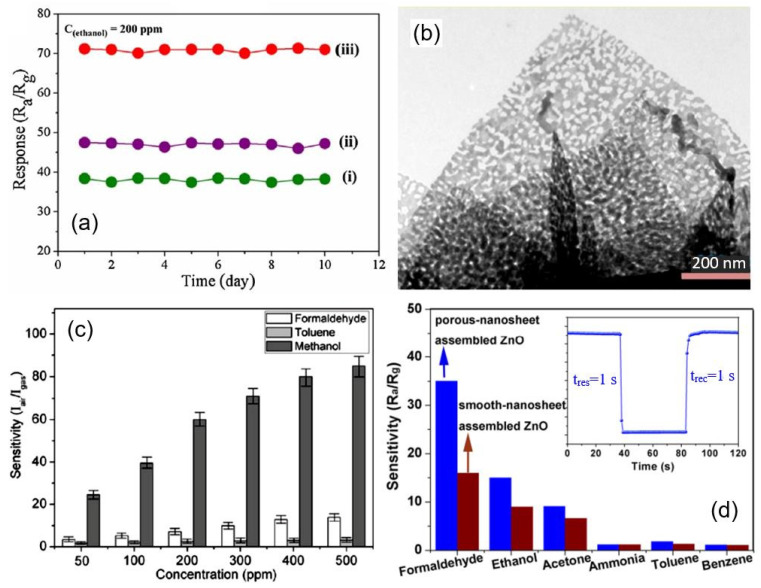
(**a**) Repeatability test of the (i) blooming, (ii) semi-blooming, and (iii) mesoporous semi-blooming SnO_2_ nanoflowers-based sensors towards 200 ppm ethanol at optimal operating temperature. (**b**) TEM images of mesoporous semi-blooming SnO_2_ nanoflowers with an average pore size of 31 nm. Reprinted with permission from Li et al. [85]. Copyright 2016: Elsevier; (**c**) Sensor responses of the gas sensor based on porous In_2_O_3_ nanoflowers toward formaldehyde, toluene, and methanol. Reprinted with permission from Liu et al. [150]. Copyright 2010: ACS; (**d**) responses of porous-nanosheet assembled ZnO and smooth-nanosheet-assembled ZnO-based sensors to 100 ppm different testing gases at 260 °C. The inset is the response transient of porous-nanosheet-assembled ZnO nanoflowers to 100 ppm HCHO at 260 °C. Reprinted with permission from Cao et al. [149]. Copyright 2017: Elsevier.

However, despite the extremely large macroporosity of such structures, which is preferable for achieving the maximum efficiency of gas sensors, the formation of pores in nanosheets and nanoflakes, even in this case, gives a positive result, which manifests itself in an increase in the sensory response (see Figure 20). Apparently, this growth occurs due to an increase in the number of active centers formed during the formation of pores [10,54]. For example, density-functional theory (DFT) calculations for porous MoO_3_ nanosheets designed to detect H_2_S show that Mo atoms at the pore edges exhibit higher adsorption activity for H_2_S and O_2_ molecules [147]. Xie et al. [148] and Zhang et al. [39] also believe that mesopores in 2D ZnO nanoflakes contributed to the increased sensitivity of sensors based on 2D ZnO nanoflowers to ethanol. Due to the porosity of the nanoflakes, successful detection of ethanol has been demonstrated down to 0.1–1 ppm. The same conclusion was reached by Cao et al. [149]. Moreover, Cao et al. [149] showed that such sensors could have a very fast response and recovery (see Figure 20d). The increased selectivity of sensors based on In_2_O_3_ nanoflakes to methanol is also explained by the presence of mesopores in [150] (see Figure 20c).

**Figure 21:**
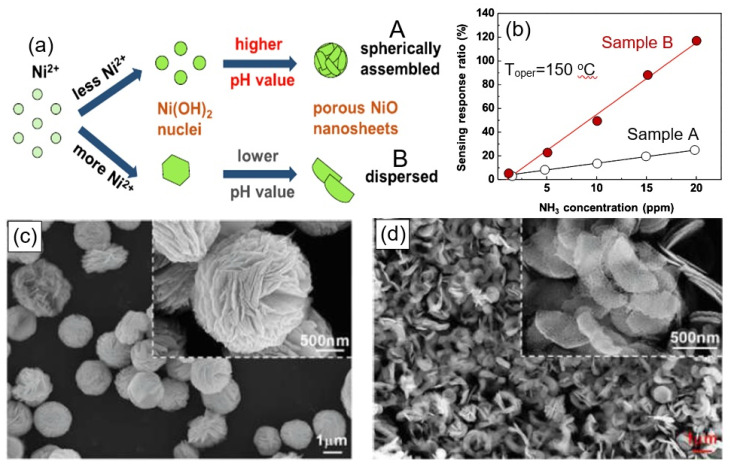
(**a**) Growing mechanism of spherically-assembled (A) and dispersed porous NiO nanosheets (B). (**b**) Sensing-response ratio to different ammonia gas concentrations for samples A and B. (**c**,**d**) SEM images of (**c**) spherically-assembled porous NiO nanosheets and (**d**) dispersed porous NiO nanosheets. Reprinted with permission from Juang et al. [151]. Copyright 2022: Elsevier.

However, it should be kept in mind that not every 3D flower-based aggregation of nanosheets is optimal for gas sensors. For example, Juang et al. [151] designed resistor-type ammonia gas sensors with two architectures of porous NiO nanosheets—spherically assembled and dispersed nanosheets (Figure 21a,c,d)— and established the devices with dispersed porous NiO nanosheets exhibited higher and faster sensing response (Figure 21b). Apparently, the spherically-assembled architecture was too dense.

**Figure 22:**
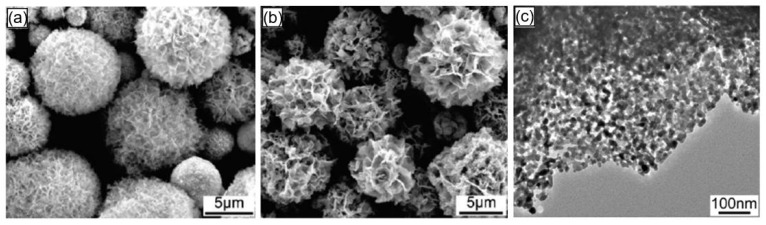
(**a**) FESEM image of the as-synthesized In_2_S_3_ precursors, (**b**) FESEM image of the products (In_2_O_3_) obtained by oxidizing In_2_S_3_ precursors. (**c**) HRTEM images of In_2_O_3_ nanoflakes. Reprinted with permission from Liu et al. [150]. Copyright 2010: ACS.

It should be noted that the porosification of NFs and NSs-based 3D structures can be organized using the same methods that were developed for the porosification of NFs and NSs and described earlier in Section 5 [150,152]. For example, Liu et al. [150] prepared a porous In_2_O_3_ nanoflower-based structure using the phase-transformation method, namely oxidation at 350–550 °C of In_2_S_3_ nanoflower synthesized via a hydrothermal route (Figure 22a). Since the radius of S atoms (0.109 nm) is larger than that of O atoms (0.065 nm), structural parameters, such as bond lengths and angles, change during the oxidation of In_2_S_3_, which manifests itself in the substitution of S by O atoms. In this condition, internal tensile stress in the crystal is formed because of the distortion of the crystal lattice. It will be released through the breakup of the crystal structure, leading to the formation of the porous structure (Figure 22c). The average pore size in such nanoflakes was around 20 nm. The same approach was used by Li et al. [153] for preparing MoO_3_ microporous nanoflowers: MoSe_2_ nanoflowers were calcined under an oxygen atmosphere at 400 °C for 3 h to be fully oxidized. Jing and Zhan [154] used annealing at 400 °C for 2 h of Zn_5_(CO_3_)_2_(OH)_6_ monoclinic hydrozincite nanoplates with an edge thickness of about 19 nm to fabricate porous ZnO nanoplates with a pore size of 20–80 nm. According to Jing and Zhan [154], during the thermal decomposition of Zn_5_(CO_3_)_2_(OH)_6_, a phase transition occurs to ZnO, in which zinc octahedra are destroyed and reorganized into tetrahedra. Therefore, they believe that the reorganization of the crystal structure and the release of water and carbon dioxide from the Zn_5_(CO_3_)_2_(OH)_6_ nanoplates are the reasons leading to the formation of holes in ZnO nanoplates and a decrease in their thickness.

**Figure 23:**
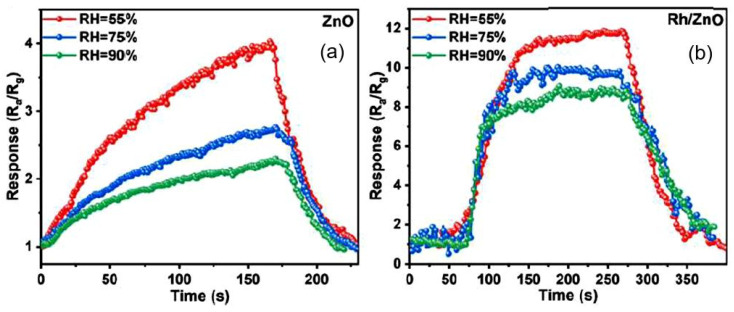
Response of (**a**) unmodified and (**b**) modified with Rh ZnO flower-based sensor to 10 ppm TMA at 220 °C under different RH. Reprinted with permission from Li et al. [153]. Copyright 2022: Elsevier.

Optimization of the parameters of gas sensors based on porous 3D flowerlike metal oxides is also carried out using the approaches that were described earlier in relation to nanoflakes and nanosheets. For example, Li et al. [153] used atomic layer deposition (ALD) of Rh onto ZnO flowerlike nanostructures to optimize sensory response to trimethylamine (TMA). The ZnO nanostructures composed of porous nanosheets were prepared by a hydrothermal method. The maximum sensory response was observed after 10 Rh deposition cycles. It was suggested that the surface modification with Rh promotes an increase in the concentration of chemisorbed oxygen on the surface of ZnO nanosheets, which is extremely important in the detection of reducing gases. The surface modification with Rh has also been found to reduce the effect of humidity on the sensors parameters (see Figure 23), which is of paramount importance for their operation in a typical atmosphere with varying humidity levels.

**Figure 24:**
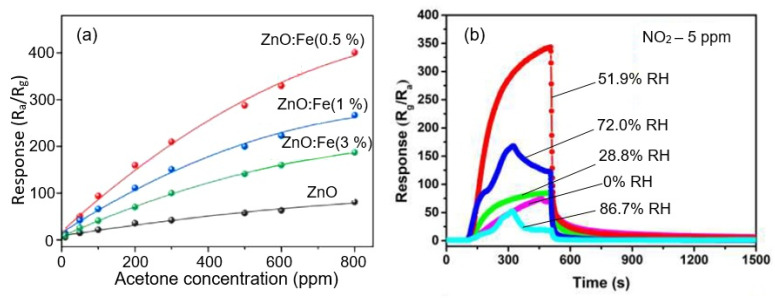
(**a**) The response of sensors based on pure ZnO and 0.5%, 1%, and 3% Fe–ZnO to 50 ppm acetone at 365 °C. Reprinted with permission from Chen et al. [155]. Copyright 2022: Elsevier; (**b**) Dynamic response curves of the In_2_O_3_/MoS_2_ sensor to 5 ppm NO_2_ in various humidity at RT. Reprinted with permission from Zhang et al. [156]. Copyright 2022: Elsevier.

Chen et al. [155] used bulk doping of ZnO with Fe (0%, 0.5%, 1%, and 3%), carried out during hydrothermal synthesis, to optimize the gas-sensitive properties of ZnO flowerlike nanostructures. They found that in order to achieve the maximum sensory response to acetone (~105 at 100 ppm) in the temperature range of 250–450 °C, the concentration of Fe should not exceed 0.5%. Otherwise, the sensory response was significantly reduced (Figure 24a). Under these conditions, the pores in the nanosheets had a maximum diameter (~20 nm), and ZnO was characterized by the maximum concentration of oxygen vacancies and the narrower band gap. As for the BET surface area, it increased with increasing Fe concentration from 21.2 m^2^/g (0%Fe) to 65.7 m^2^/g (3%Fe) and was not a factor determining the magnitude of the sensory response. Zhang et al. [156], to optimize the conductometric response of 3D In_2_O_3_ microflower-based sensors to NO_2_, proposed to modify the surface of porous In_2_O_3_ nanosheets with MoS_2_. The results showed that the In_2_O_3_/MoS_2_ composite-based sensor at room temperature exhibits a response as high as 343 at 5 ppm NO_2_, which is 309 and 73 times higher than the response of the sensors based on the pristine MoS_2_ and In_2_O_3_. In addition, the sensors had better selectivity to NO_2_. Improving the sensory properties of the In_2_O_3_/MoS_2_ composite-based sensor, Zhang et al. [156] explained by (i) the presence of an electron depletion layer at the interface of In_2_O_3_ and MoS_2_, (ii) the highly exposed edges of MoS_2_ to provide more active sites, and (iii) 2D/3D hybrid structure to improve the adsorption-desorption process of NO_2_. However, one should always keep in mind that the improvement of some properties may be accompanied by the deterioration of others [37,157]. In particular, it turned out that the In_2_O_3_/MoS_2_ composite-based sensor exhibited increased sensitivity to air humidity (Figure 24b). This naturally complicates the use of such sensors since in order to avoid the effect of humidity on the sensor, it is necessary either to first remove moisture from the gases or to use a complex electronic system to compensate for the effect of humidity.

**Figure 25:**
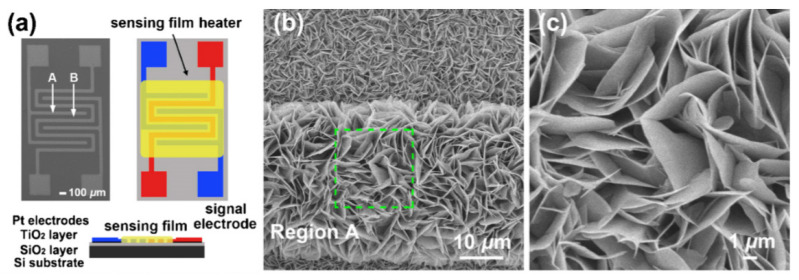
(**a**) Schematic diagrams of the different views for the sensor platform; (**b**,**c**) FE-SEM images of ZnO nanosheets (NSs) having a 3D structure taken from different regions in the sensor platform. Reprinted with permission from [161]. Copyright 2012: Elsevier.

Despite the macroporosity of flowerlike structures, it is believed that the most optimal 3D nanoflakes-based structure for developing gas sensors is still the structure with vertical walls shown in Figure 25b. In such structures, unlike nanoflower-based structures, there is no nonporous core, which may not participate in gas-sensing effects. Studies have shown that the structures can show the highest sensitivity and response rate when interacting with a gas environment [158,159]. At nanoflakes thicknesses comparable with the Debye length, the change in the resistance of a gas-sensitive layer having such a structure will be controlled not only by the resistance of inter-nanoflakes contacts but also by the resistance of the nanoflakes themselves. In particular, if the thickness of the nanosheets of n-type conductivity is less than twice the Debye length, then the whole nanosheet body will be almost electron-depleted in air, which will result in significantly enhanced sensor response based on the grain-control model [60] during interaction with reducing gases. As known, reducing gases reduce surface band bending due to interaction with chemisorbed oxygen [111]. When detecting oxidizing gases, this condition is unacceptable since the sensory response will be insignificant in this case. Under these conditions, an increase in the surface potential caused by interaction with oxidizing gases [112] will not be accompanied by a noticeable increase in the resistance of NSs and NFs. To achieve a significant effect in this case, the thickness of NFs and NSs must be greater than twice the Debye length, and complete blocking of the current-flow path through NFs and NSs must be observed only when an oxidizing gas appears (see Figure 3). Experimental confirmation of the influence of the thickness of porous nanosheets and nanoplates on the sensor response can be found in [160] in relation to ZnO-based gas sensors.

Gas-sensitive layers with the structure shown in Figure 25b are usually grown directly on the surface of the platform used to fabricate the gas sensor [161,162,163,164]. For example, in [162,163,164], NiO, Co_3_O_4,_ and CuO nanosheets were synthesized directly on an Al_2_O_3_ tube, inside which a heater was subsequently mounted, and in [161,164], ZnO and NiO nanosheets were synthesized directly on a substrate with already-formed contacts (see Figure 25a). However, the technology proposed in these works is poorly consistent with large-scale sensor production. If such structures are synthesized separately and then attempted to be transferred to a sensor platform, then the probability of preserving such structures in the gas-sensitive layer is minimal. In this regard, 3D structures in the form of nanospheres and nanoflowers are more likely to retain their structure in manufacturing sensors based on the principles of thick-film technology. Therefore, it must be recognized that, despite the progress made, the efficient integration of 2D nanoflakes and nanosheets, especially in the form of 3D structures, with a sensor platform remains a serious problem that limits the use of such nanostructures in the development of gas sensors [165].

In addition, it must be borne in mind that excessive porosity can cause a decrease in the mechanical strength of the gas-sensitive layer. Therefore, the process of nanoflakes and nanosheet porosification must be optimized to achieve a porosity that provides maximum sensor response while maintaining the mechanical strength and stability of the formed gas-sensitive layer. This primarily applies to 3D structures intended to manufacture gas sensors using thick-film technology. Unfortunately, research in this direction has not yet been carried out.

## 8. Summary

Our analysis shows that the porosification of 2D materials, such as nanoflakes and nanosheets, is an effective way to improve the performance of gas sensors. However, the use of porous structures, in addition to complicating the process of manufacturing gas-sensitive materials, introduces additional uncertainty in achieving the required sensor parameters. Sensor parameters become dependent not only on the structural and electrophysical parameters of 2D nanoflakes and nanosheets but also on the density of nanopores in NSs and NFs and their diameter, which will certainly be accompanied by a decrease in the reproducibility of the parameters of sensors based on such materials. However, we did not find any studies aimed at establishing the influence of pore parameters in nanoflakes and nanosheets on gas-sensitive effects and sensor parameters. This can become a significant obstacle to using porous 2D structures in developing gas sensors intended for the market. The lack of systematic studies aimed at a more detailed and deep understanding of the relationship between synthesis conditions, the structure of 2D nanoflakes, and the performances of gas sensors based on such materials also hinder progress in this direction. Unfortunately, research in this area is mostly demonstrative—what is obtained as a result of synthesis is then tested without proper optimization of the process and attempts to understand why these results were obtained and what needs to be done to improve them.

## Data Availability

Not applicable.

## Supplementary Materials

The following supporting information can be downloaded at: https://www.mdpi.com/article/10.3390/nano13020237/s1, Table S1: Parameters of gas sensors based on porous 2D metal oxide nanostructures.
