# Supplementary material for: Current Trends in Nanomaterials for Metal Oxide-Based Conductometric Gas Sensors: Advantages and Limitations—Part 2: Porous 2D Nanomaterials
Source: Nanomaterials (Basel). 2023 Jan 5;13(2):237. doi: 10.3390/nano13020237 (PMC9867534; doi:10.3390/nano13020237)
Supplement: Supplementary file 1 [file nanomaterials-13-00237-s001.zip › nanomaterials-2124612-SI.pdf]

Supplementary files

Table S1. Parameters of gas sensors based on porous 2D metal oxide nanostructures

Material (Form) (Technology)	GST	STR	T, nm	PD, µm	PS, nm	T_{oper}, °C	Target gas	C_{gas}, ppm	Response	DL, ppm	Res./ Rec. time, s	Ref.
SnO_2 (NF) (SnS_2 calcination)	DC	3D	~30	0.5-1.0	~18	220	Ethanol	100	R_{air}/R_{gas} ~ 7	<5	8/17	1
SnO_2 (NF) (HT)	CT	3D	N/A	N/A	N/A	200	Ethanol	200	R_{air}/R_{gas} ~46	<20	6/5	2
SnO_2:CeO_2 (NS) (HT)	CT	2D	15-30	0.8-1.0	20-30	340	Ethanol	100	R_{air}/R_{gas} ~44	<5	25/6	3
SnO_2:CeO_2 (NS)	CT	2D	N/A	10-15	10-15	160	3H-2B	50	R_{air}/R_{gas} ~620	~1	29/172	4
SnO_2:NiO(5%) (NS) (HT)	CT	3D	10-20	1-2	3-4	100	HCHO	100	R_{air}/R_{gas} ~39	<5	28/115	5
SnO_2 (NS) (HT)						200			R_{air}/R_{gas} ~11		53/99	
SnO_2 (NS) (HT)	CT	3D	N/A	~2	27	340	Acetic acid	100	R_{air}/R_{gas} ~132	<20	11/6	6
WO_3 (NF) (LA)	DC	2D	~50	1.5-3.5	~20	250	Ethanol	100	R_{air}/R_{gas} ~ 3	<10	N/A	7
WO_3:Pd (NF) (ST)	ISG	3D	20-40	~1	4-100	200	NO	20	R_{gas}/R_{air} ~200	<1	135/50	8
WO_3 (NS) (CBD)	ISG	3D	20	N/A	N/A	100	NO_2	10	R_{gas}/R_{air} ~ 460	< 1	54/63	9
WO_3–rGO(20%) (NS) (HT)	CT	2D	N/A	0.3-0.5	N/A	90	NO_2	10	R_{gas}/R_{air} ~4	1-2	10/9	10
ZnO (NF) (HT)	CT	2D	N/A	N/A	N/A	350	Ethanol	50	R_{air}/R_{gas} ~ 22	N/A	12/14	11
		3D		1-2					R_{air}/R_{gas} ~ 87		7/9	
ZnO (NF) (PM)	ISG	3D	~9	0.4-0.7	3-50	250	Ethanol	50	R_{air}/R_{gas} ~ 37	<2	14/8	12
ZnO (NS) (HT)	CT	2D	~100	N/A	~80	400	Ethanol	100	R_{air}/R_{gas} ~11	<10	12/231	13
ZnO (NS) (CS)	CT	3D	12-20	3-5	~5	320	Ethanol	100	R_{air}/R_{gas} ~24	< 1	3/15	14
ZnO (NS) (HT)	CT	3D	10-40	3-6	9-45	400	Ethanol	200	R_{air}/R_{gas} ~400	~0.0.1	N/A	15
ZnO (NS) (ST)	CT	3D	20-40	1-2	7-25	280	Ethanol<br>Acetone	100	R_{air}/R_{gas} ~24<br>R_{air}/R_{gas} ~32	<5	6/23<br>5/28	16
ZnO (NF) (HT)	CT	3D	20	1-3	3-40	420	Acetone	100	R_{air}/R_{gas} ~17	<5	9/8	17
ZnO (NS) (HT)	ISG	3D	N/A	~0.5	2-10	400<br>400<br>200	Ethanol<br>Acetone<br>H_2S	50<br>50<br>2.5	R_{air}/R_{gas} ~5<br>R_{air}/R_{gas} ~16<br>R_{air}/R_{gas} ~ 163	9/22<br>9/22<br>3/43	~2<br><1<br><1	18
ZnO (NS) (CS)	CT	2D	2.9-3.4	N/A	N/A	340	$C_5H_8O_2$	100	R_{air}/R_{gas}~190	~ 1	19/94	19
ZnO (NF) (MHT)	CT	3D	40-80	N/A	30-60	170	NO_2	0.5	R_{gas}/R_{air} ~ 125	<0.1	N/A	20
ZnO (NS) (ST)	DC	2D	80	~1	60	200	NO_2	0.01	R_{gas}/R_{air} ~75	~0.1	N/A	21
ZnO (NS) (HT)	SP	2D	~15	0.5	~26	250	NH_3	100	R_{air}/R_{gas} ~6	< 10	8/17	22

							HCHO		R_{air}/R_{gas} ~4		13/22	
ZnO (NS) (HT)	CT	3D	30-80	1-2	~40	370	DMA	200	R_{air}/R_{gas} ~2000	<1	16/15	23
ZnO:Pd (NS) (CS+R)	ISG	3D	N/A	~1	N/A	220	CO	100	R_{air}/R_{gas} ~15	20	100/255	24
ZnO (NS) (CS)	CT	2D	N/A	1-2	14	320	Isopropanol	100	R_{air}/R_{gas} ~10	~1	N/A	25
ZnO:CdS (NS) (CS)					47				R_{air}/R_{gas} ~33	N/A		
In_2O_3 (NS) (ST)	CT	3D	20	1-2	~50	140	NO_2	0.2	R_{gas}/R_{air} ~ 40	<0.01	N/A	26
In_2O_3 (NS) (HT)	CT	3D	15-30	1-2	5-25	RT	NO_2	0.5	R_{gas}/R_{air} ~620	N/A	600/900	27
In_2O_3 (NS) (HT)	CT	2D	~4	0.5	~4	120	NO_x	10	R_{gas}/R_{air} ~213	~0.01	4/10	28
In_2O_3 (NS) (HT)	CT	3D	~3	~1	3-5	270	Ethanol	100	R_{air}/R_{gas} ~ 66	~0.5	12/10	29
In_2O_3:Ag,Pd (NS) (CS)	CT	3D	N/A	3.5-5	2-50	180	Toluene,	1	R_{air}/R_{gas} ~ 16	~0.02	7/13	30
In_2O_3:Yb (NS) (ST)	CT	2D	23	0.2	N/A	220	Acetone	50	R_{air}/R_{gas} ~15	<5	<10/<10	31
Co_3O_4 (NS) (HT)	CT	2D	30	3-5	~3	150	Acetone	100	R_{air}/R_{gas} ~ 11	<10	N/A	32
Co_3O_4:Mn (NS) (ST)	CN	2D	N/A	N/A	2.3	125	CO	40	R_{air}/R_{gas} ~ 3.8	0.5	12/26	33
Co_3O_4:CdS (NS)	ISG	3D	3	1	2-100	RT	H_2S	100	R_{air}/R_{gas} ~13	1-5	0.6/1	34
CuO(NS) (HT)	ISG	2D	62	0.5-1.2	5-17	RT	H_2S	0.01	R_{air}/R_{gas} ~1.25	N/A	234/76	35
CeO_2 (NS) (HT-D)	N/A	2D	N/A	N/A	~15	450	CO	500	R_{air}/R_{gas} ~1.12	~10	2/2	36
TiO_2:Pd (NS) (TM)	CT	2D	N/A	1-10	3-8	230	H_2	1000	R_{air}/R_{gas} ~9	~1	1.6/1.4	37
CuO (NS) (HT-D) c-CuO	CT	3D	N/A	~1	15-30	260	Ethanol<br>HCHO	100	R_{air}/R_{gas} ~15<br>R_{air}/R_{gas} ~11	< 1	13/8<br>15/17	38
a-CuO							Ethanol<br>HCHO	50	R_{air}/R_{gas} ~40<br>R_{air}/R_{gas} ~50	<0.1	11/6<br>8/16	
CuO(NF)-rGO (HT)	DC	2D	20-40	>1	2-2.5	23	NO_2	100	R_{air}/R_{gas} ~150	<0.05	7/50	39
NiO (NS) (SCS)	ISG	2D	N/A	N/A	N/A	200	Ethanol	100	R_{air}/R_{gas} ~ 2	<10	N/A	40
NiO (NS) (HT)	DC	3D	N/A	0.3-0.35	~5	150	NH_3	15	R_{air}/R_{gas}~1.9	<2	140/70	41
NiO (NS)	ISG	3D	<10	<1	1.5-15	225	Acetone	0.1	R_{air}/R_{gas}~6	<0.01	N/A	42
NiO (NS) (HT)	CT	2D	~15-20	0.2-0.4	~90	92	N_2H_4	100	R_{air}/R_{gas}~110	~0.01	50/30	43
V_2O_5-NiO (NS) (CS)	SP	3D	~5	~20	N/A	200	H_2S	0.5	R_{air}/R_{gas} ~6.5	0.02	8/25	44
NiO:Fe_2O_3(1.5%) (NS) (CS-D)	CT	3D	N/A	N/A	~23	255	Ethanol<br>Methanol	100	R_{air}/R_{gas} ~170<br>R_{air}/R_{gas} ~108	<5	0.5/15<br>0.1/11	45

$C_5H_8O_2$ – acetylacetone; CBD – chemical bath deposition; C(gas) – gas concentration; CS - chemical synthesis; CS+R - chemical synthesis+ reduction; CT - ceramic technology; DC - drop-casting method; DL – detector limit; DMA - dimethylamine; GST – gas sensor technology; 3H-2B - 3-hydroxy-2-butanone; HCHO – formaldehyde; HT –hydrothermal; HT-D – hydrothermal synthesis +decomposition; ISG - in-situ growth; LA – pulse laser ablation; MHT – microwave hydrothermal; N/A – not available; NF- nanoflakes; NS – nanosheets; PD - planar dimension; PM – precipitation method; PS - pore size; rGO – reduced graphene

oxide; RT – room temperature; SC - sonochemical method; SCS - solution combustion synthesis; SP - screen-printing; ST – solvothermal process; STR – structure; T - thickness; TM – template method;

References

1. Q. Wang, D. Wang, M. Wu, B. Liu, J. Chen, T. Wang, J. Chen, Porous SnO_2 nanoflakes with loose-packed structure: Morphology conserved transformation from SnS2 precursor and application in lithium ion batteries and gas sensors. J. Phys. Chem. Solids 72 (2011), 630-636.

2. O. Almamoun, S. Ma, A. Alshiply, Facile Synthesis of 3D Porous SnO_2 Nano Flakes by a Simple Hydrothermal Method and Their Ethanol Gas Sensor Properties. Intern. J. Eng. Res. Manag. (IJERM) 5 (3) (2018), 25-28.

3. S. Yan, X. Liang, H. Song, S. Ma, Y. Lu, Synthesis of porous CeO_2-SnO_2 nanosheets gas sensors with enhanced sensitivity. Ceram Intern. 44 (2018), 358-363.

4. X.-Y. Yang, Y.-T. Shi, F.-L. Gong, J.-L. Chen, G.-X. Jin, Q. Guo, et al., Asymmetric interfacial oxygen sites of porous CeO_2-SnO_2 nanosheets enabling highly sensitive and selective detection of 3-hydroxy-2-butanone biomarkers. Sens. Actuators B 371 (2022), 132500.
5. D. Meng, D. Liu, G. Wang, Y. Shen, X. San, M. Li, F. Meng, Low-temperature formaldehyde gas sensors based on NiO-SnO_2 heterojunction microflowers assembled by thin porous nanosheets. Sens. Actuators B 273 (2018) 418-428.

6. T.T. Wang, S.Y. Ma, L. Cheng, X.L. Xu, J. Luo, X.H. Jiang, W.Q. Li, W.X. Jin, X.X. Sun, Performance of 3D SnO_2 microstructure with porous nanosheets for acetic acid sensing. Mater. Lett. 142 (2015) 141-144.

7. J. Xiao, P. Liu, Y. Liang, H. B. Li and G. W. Yang, Porous tungsten oxide nanoflakes for highly alcohol sensitive performance Porous tungsten oxide nanoflakes for highly alcohol sensitive performance Porous tungsten oxide nanoflakes for highly alcohol sensitive performance. Nanoscale, 2012, 4, 7078-7083.

8. Z.-X. Cai, H.-Y. Li, J.-C. Ding, X. Guo, Hierarchical flowerlike WO_3 nanostructures assembled by porous nanoflakes for enhanced NO gas sensing. Sens. Actuators B 246 (2017) 225-234.

9. M. Wang, Y. Wang, X. Li, C. Ge, S. Hussain, G. Liu, G. Qiao, WO_3 porous nanosheet arrays with enhanced low temperature NO_2 gas sensing performance. Sens. Actuators B 316 (2020), 128050.

10. Q. Hao, T. Liu, J. Liu, Q. Liu, X. Jing, H. Zhang, G. Huang, J. Wang, Controllable synthesis and enhanced gas sensing properties of a single-crystalline WO_3–rGO porous nanocomposite. RSC Adv. 7 (2017), 14192-14199.

11. W. Guo, T. Liu, J. Wang, W. Yu, R. Sun, Y. Chen, S. Hussain, X. Peng, Z. Wang, Hierarchical ZnO porous microspheres and their gas-sensing properties Hierarchical ZnO porous microspheres and their gas-sensing properties. Ceram. Intern. 39 (2013) 5919-5924.

12. F. Fan, P. Tang, Y. Wang, Y. Feng, A. Chen, R. Luo, D. Li, Facile synthesis and gas sensing properties of tubular hierarchical ZnO self-assembled by porous nanosheets. Sens. Actuators B 215 (2015) 231-240.

13. L.V. Duy, N.H. Hanh, D.N. Son, P.T. Hung, C.M. Hung, N.V. Duy, et al., Facile Hydrothermal Synthesis of Two-Dimensional Porous ZnO Nanosheets for Highly Sensitive Ethanol Sensor. J. Nanomater. 2019, 4867909.

14. J. Huang, Y. Wu, C. Gu, M. Zhai, Y. Sun, J. Liu, Fabrication and gas-sensing properties of hierarchically porous ZnO architectures. Sens. Actuators B 155 (2011) 126-133.

15. L. Zhang, J. Zhao, H. Lu, L. Li, J. Zheng, H. Li, Z. Zhu, Facile synthesis and ultrahigh ethanol response of hierarchically porous ZnO nanosheets. Sens. Actuators B 161 (2012) 209-215.

16. J. Li, H. Fan, X. Jia, Multilayered ZnO Nanosheets with 3D Porous Architectures: Synthesis and Gas Sensing Application. J. Phys. Chem. C 114 (2010), 14684–14691.

17. X. Wang, W. Liu, J. Liu, F. Wang, J. Kong, S. Qiu, C. He, L. Luan, Synthesis of Nestlike ZnO Hierarchically Porous Structures and Analysis of Their Gas Sensing Properties. ACS Appl. Mater. Interfaces 4 (2012), 817−82

18. Y. Zhu, Y. Wang, G. Duan, H. Zhang, Y. Li, G. Liu, L. Xu, W. Cai, In situ growth of porous ZnO nanosheet-built network film as high-performance gas sensor. 221 (2015), 350-356.

19. F. Liu, X. Wang, X. Chen, X. Song, J. Tian, H. Cui, Porous ZnO Ultrathin Nanosheets with High Specific Surface Areas and Abundant Oxygen Vacancies for Acetylacetone Gas Sensing. ACS Appl. Mater. Interfaces 11 (2019), 24757−24763.

20. M. Chen, Z. Wang, D. Han, F. Gu, G. Guo, Porous ZnO Polygonal Nanoflakes: Synthesis, Use in High-Sensitivity NO_2 Gas Sensor, and Proposed Mechanism of Gas Sensing. J. Phys. Chem. C 2011, 115, 12763–12773.

21. M.S. Choi, M.Y. Kim, A. Mirzaei, H.-S. Kim, S.-I. Kim, S.-H. Baek, et al., Selective, sensitive, and stable NO_2 gas sensor based on porous ZnO nanosheets. Appl. Surf. Sci. 568 (2021) 150910.

22. J. Liu, Z. Guo, F. Meng, T. Luo, M. Li, J. Liu, Novel porous single-crystalline ZnO nanosheets fabricated by annealing $ZnS(en)_{0.5}$ (en = ethylenediamine) precursor. Application in a gas sensor for indoor air contaminant detection. Nanotechnology 20 (2009) 125501.

23. L. Zhang, J. Zhao, H. Lu, L. Li, J. Zheng, J. Zhang, H. Li, Z. Zhu, Highly sensitive and selective dimethylamine sensors based on hierarchical ZnO architectures composed of nanorods and nanosheet-assembled microspheres. Sens. Actuators B 171-172 (2012b) 1101-1109.

24. N. Luo, B. Zhang, D. Zhang, J. Xu, Enhanced CO sensing properties of Pd modified ZnO porous nanosheets. Chinese Chem. Lett. 31 (2020) 2033-2036.

25. H. Zhang, Z. Jin, M.-D. Xu, Y. Zhang, J. Huang, H. Cheng, et al., Enhanced isopropanol sensing performance of the CdS nanoparticle decorated ZnO porous nanosheets based gas sensors. IEEE Sensors J. 21 (12) (2021a), 13041-13047.

26. X. Xu, X. Li, H. Zhang, C. Feng, C. Wang, F. Liu, Synthesis, characterization and gas sensing properties of porous flower-like indium oxide nanostructures. RSC Adv. 5 (2015), 30297-30302.

27. X. Xu, D. Wang, W. Wang, P. Sun, J. Ma, X. Liang, et al., Porous hierarchical In_2O_3 nanostructures: Hydrothermal preparation and gas sensing properties. Sens. Actuators B 171-172 (2012a), 1066-1072.

28. X. Wang, J. Su, H. Chen, G.-D. Li, Z. Shi, H. Zou, X. Zou, Ultrathin In_2O_3 nanosheets with uniform mesopores for highly sensitive nitric oxide detection. ACS Appl. Mater. Interfaces 9 (19) (2017), 16335–16342.

29. W. Qin, Z. Yuan, H. Gao, F. Meng, Ethanol Sensors Based on Porous In_2O_3 Nanosheet-Assembled Micro-Flowers. Sensors 2020, 20, 3353;

30. X. Liu, X. Duan, C. Zhang, P. Hou, X. Xu, Improvement toluene detection of gas sensors based on flower-like porous indium oxide nanosheets. J. Alloys Compounds 897 (2022), 163222.

31. W.H. Zhang, S.-J. Ding, Q.-S. Zhang, H. Yi, Z.-X. Liu, M.-L. Shi, R.-F. Guan, L. Yue, Rare earth element-doped porous In_2O_3 nanosheets for enhanced gas-sensing performance. Rare Met. 40(6) (2021b), 1662–1668.

32. Z. Zhang, Z. Wen, Z. Ye, L. Zhu, Gas sensors based on ultrathin porous Co_3O_4 nanosheets to detect acetone at low temperature. RSC Adv. 5 (2015), 59976-59982.

33. C. Qin, B. Wang, Y. Wang, Metal-organic frameworks-derived Mn-doped Co_3O_4 porous nanosheets and enhanced CO sensing performance. Sens. Actuators B 351 (2022) 130943.

34. M. Dun, J. Tan, W. Tan, M. Tang, X. Huang, CdS quantum dots supported by ultrathin porous nanosheets assembled into hollowed-out Co_3O_4 microspheres: A room-temperature H_2S gas sensor with ultra-fast response and recovery. Sens. Actuators B 298 (2019) 126839.

35. Z. Li, N. Wang, Z. Lin, J. Wang, W. Liu, K. Sun, Y.Q. Fu, Z. Wang, Room-Temperature High-Performance H_2S Sensor Based on Porous CuO Nanosheets Prepared by Hydrothermal Method. ACS Appl. Mater. Interfaces, 8 (32) (2016), 20962-20968.

36. P. Li, B. Wang, Y. Wang, Ultrafast CO Sensor Based on Flame-annealed Porous CeO_2 Nanosheets for Environmental Application. J. Inorg. Mater. 36 (11) (2021), 1223-1230.

37. D. Wang, J. Yang, L. Bao, Y. Cheng, L. Tian, Q. Ma, et al., Pd nanocrystal sensitization two-dimension porous TiO_2 for instantaneous and high efficient H_2 detection. J. Colloid Interface Sci. 597 (2021) 29-38.

38. Z. Tian, H. Bai, Y. Li, W. Liu, J. Li, Q. Kong, G. Xi, Gas-Sensing Activity of Amorphous Copper Oxide Porous Nanosheets. ChemistryOpen 9 (2020), 80–86.

39. H. Bai, H. Guo, J. Wang, Y. Dong, B. Liu, Z. Xie, et al., A room-temperature NO2 gas sensor based on CuO nanoflakes modified with rGO nanosheets. Sens. Actuators B 337 (2021), 129783.

40. C. Dong, X. Xiao, G. Chen, H. Guan, Y. Wang, I. Djerdj, Porous NiO nanosheets self-grown on alumina tube using a novel flash synthesis and their gas sensing properties. RSC Adv. 5 (2015), 4880-4885.

41. F.-R. Juang, C.-H. Hsieh, I.-Y. Huang, W.-Y. Wang, W.-B. Lin, L. Yen, Dispersed and spherically assembled porous NiO nanosheets for low concentration ammonia gas sensing applications. Solid State Electron. 189 (2022) 108224.

42. C. Li, P.G. Choi, K. Kim, Y. Masuda, High performance acetone gas sensor based on ultrathin porous NiO nanosheet. Sens. Actuators B 367 (2022), 132143.

43. S. Feng, H. Yu, X. Zhang, L. Huo, R. Gao, P. Wang, et al., Ionic liquid-assisted synthesis of 2D porous lotus root slice-shaped NiO nanomaterials for selective and highly sensitive detection of N2H4. Sens. Actuators B 359 (2022) 131529.

44. T. Qiu, N. Luo, M. Guo, H. Cai, Z. Cheng, J. Xu, Synthesis and Enhanced H_2S Sensing Properties of V_2O_3 -NiO Nanoflower Assembled by Porous Nanosheets. J. Electrochem. Soc. 169 (2022), 037504.

45. W. Tan, J. Tan, L. Fan, Z. Yu, J. Qian, X. Huang, Fe_2O_3-loaded NiO Nanosheets for Fast Response/recovery and High Response Gas Sensor. Sens. Actuators B 256 (2018) 282-293.
